# A Two-Step Estimation Approach of Latent Class-Item Response Theory Models in the Presence of DIF and Multidimensionality

**DOI:** 10.1177/00131644261467757

**Published:** 2026-08-21

**Authors:** Zsuzsa Bakk, Rosa Fabbricatore

**Affiliations:** 1 Leiden University, The Netherlands; 2 University of Naples Federico II, Naples, Italy

**Keywords:** latent class IRT model, two-step estimation, DIF, multidimensional model

## Abstract

Estimation of Latent class item response theory (LC-IRT) models is typically performed using full-information maximum likelihood, which may be computationally demanding, susceptible to convergence problems, and subject to interpretational confounding. We propose a two-step estimation approach for unidimensional and multidimensional LC-IRT models with covariates and differential item functioning (DIF). In the first step, the measurement model is estimated, including direct covariate effects on item responses when DIF is part of the final measurement specification. In the second step, covariate effects on latent class membership are estimated while treating the measurement parameters as fixed. We evaluate the two-step estimator through simulation studies and identify the conditions under which it yields low bias, particularly when measurement quality and entropy are adequate. We then apply the proposed approach to data on student performance in statistics.

## Introduction

Latent class item response theory (LC-IRT) models are powerful tools for analyzing categorical response data, allowing researchers to uncover unobserved heterogeneity in populations (Mislevy & Verhelst, 1990; Rost, 1990). These models combine the strengths of latent class analysis and item response theory, providing a nuanced understanding of latent traits across distinct subpopulations. Applications of the model include diagnosing subgroups of skills for students, such as in language learning (Noroozi & Karami, 2026; Roussos et al., 2007; Sen & Cohen, 2019; Tolar et al., 2021), or for risky youth behavior (Finch and Pierson 2011) or more methodological applications identifying forms of differential item functioning (DIF) in IRT modeling (Choi et al., 2015; Tijmstra et al., 2018). Further applications can be found in political science, for example, to identify forms of citizenship in governance structures (Chow & Kennedy, 2012). Sen and Cohen (2019) examined the use of LC-IRT models on a large time frame from 1995 to 2018 and found that the approach was most often used to model differentiated learning profiles in mathematics (32% of the papers they investigated), personality assessment (20%) and investigation of latent DIF (16%).

Despite their popularity, LC-IRT models are challenging to estimate. The standard approach—full information maximum likelihood (FIML)—requires simultaneous estimation of the measurement and structural components, often resulting in convergence problems and high computational burden, particularly in multidimensional models (Chen et al., 2017; Rijmen, 2009). Moreover, FIML estimation with covariates may lead to interpretational confounding, whereby relationships with covariates alter the meaning of the latent variables themselves (Asparouhov & Muthén, 2014a; Bakk & Kuha, 2018; Vermunt, 2010). These limitations motivate the search for alternative estimation strategies that are both computationally efficient and conceptually transparent.

Stepwise estimation methods offer such an alternative. In these approaches, the measurement model is estimated first, followed by the structural model in a subsequent step, with appropriate correction for classification or measurement error. Stepwise estimators are now well established in latent class analysis and structural equation modeling (SEM), including robust three-step methods (Asparouhov & Muthén, 2014a; Vermunt, 2010), two-step approaches (Bakk & Kuha, 2018), and related frameworks such as structural-after-measurement models in SEM (Rosseel & Loh, 2024). A unifying principle in all of these methods is the deliberate separation of the measurement and structural components, which reduces computational complexity and protects against interpretational confounding (Vermunt, 2025). Vermunt (2025) argues that stepwise approaches can be separated into three main types, namely fixed parameters approaches, single indicator approaches, and bias correction approaches. In what follows, we focus on the fixed-parameters approach, also known as the two-step approach, as it seems to offer the most flexibility for extension to the LC-IRT framework.

Recent methodological work has demonstrated that stepwise estimators can achieve performance comparable to FIML under favorable conditions, while offering greater robustness and scalability. Bakk and Kuha (2018) show that for latent class models the two-step estimators perform well when classification uncertainty is limited and provide clear guidance on efficiency–robustness trade-offs. Extensions have been developed for multilevel latent class models (Lyrvall et al., 2025), longitudinal latent Markov models (Di Mari & Bakk, 2018), and similar two-step estimators were also proposed for the general SEM framework (Rosseel & Loh, 2024).

Applying stepwise estimation to LC-IRT models is a natural next step. LC-IRT models are particularly prone to estimation difficulties when extended with covariates, DIF effects, or multidimensional latent structures. Two-step estimation allows researchers to first establish and interpret the latent class–IRT measurement model, and only then examine covariate effects on classes or dimensions. This modularity simplifies estimation, improves convergence, and facilitates flexible modeling of DIF without inflating model complexity (Di Mari & Bakk, 2018; Vermunt & Magidson, 2021).

These advantages are especially relevant for multidimensional LC-IRT models, where multiple latent traits further increase computational demands and the risk of interpretational confounding under FIML estimation. Stepwise approaches offer a stable alternative by disentangling the recovery of latent profiles from their associations with external variables (Bakk & Kuha, 2018).

In this article, we propose a two-step estimator for LC-IRT models, covering both unidimensional and multidimensional settings. We evaluate the performance of the proposed estimator through simulation studies and an empirical application. Our results demonstrate that the two-step approach provides a computationally efficient and interpretable alternative to FIML, while maintaining robust parameter recovery in complex LC-IRT models.

The remainder of the paper is organized as follows. We first introduce the LC-IRT model with covariates and describe the proposed two-step estimation procedure. Next, we present simulation studies focusing on models with DIF and multidimensional latent structures. We then illustrate the approach using an empirical application on students’ statistical skills. Finally, we conclude with a discussion and directions for future research.

## Two-Step Estimation of Latent Class IRT Models With Covariates

### Model Specification

LC-IRT models (Mislevy & Verhelst, 1990; Rost, 1990) integrate continuous latent traits and categorical latent variables to capture both quantitative and qualitative unobserved heterogeneity in item response data. Consider a sample of n independent respondents indexed by i=1,…,n . Each respondent provides responses to a set of K categorical indicators, denoted by the vector $Y_{i}=\left(Y_{i1},\ldots ,Y_{\mathrm{iK}}\right)$ , where each item $Y_{\mathrm{ik}}$ takes values in $\left\{0,1,\ldots ,m_{k}\right\}$ . We assume that the K indicators measure the latent trait $\theta_{i}$ , which represents the unobserved ability or characteristic of individual i . Standard IRT models typically assume that $\theta_{i}$ follows a Gaussian density function $\theta_{i}\;\sim \;N\left(0,1\right)$ and that the entire sample belongs to this qualitatively homogeneous distribution. The mixture IRT framework relaxes this homogeneity assumption by introducing a latent categorical variable $X_{i}$ with C unordered classes, $X_{i}\in \{1,\ldots ,C\}$ , representing unobserved subpopulations differing in their item and/or person parameters. Moreover, mixture IRT models relax the assumption of traditional latent class models on equal item response probabilities for all individuals within a latent class, simultaneously estimating both individual latent trait levels and latent class membership. Accordingly, the probability of observing a specific response pattern $Y_{i}$ is defined as follows:

(1) $$P\left(Y_{i}\right)=\sum_{c=1}^{C}\pi_{c}\int_{\Theta_{c}}\left[\overset{K}{\underset{k=1}{\Pi }}P\left(Y_{\mathrm{ik}}|\theta_{i},X_{i}=c\right)\right]f_{c}\left(\theta_{i}\right)d\theta_{i}$$

where $\pi_{c}$ denotes the class proportion, with constraints $0\leq \pi_{c}\leq 1$ and $\sum \pi_{c}=1$ ; $f_{c}\left(\theta_{i}\right)$ is the class-specific latent trait distribution, typically $\theta_{i}|X_{i}=c\;\sim \;N\left(\mu_{c},\sigma_{c}^{2}\right)$ , allowing latent classes to differ not only in their item parameters but also in the location and dispersion of the underlying trait. The item response probabilities $P\left(Y_{\mathrm{ik}}|\theta_{i},X_{i}=c\right)$ satisfy the assumption of local independence, such that item responses are conditionally independent given the latent trait $\theta_{i}$ and the latent class membership $X_{i}=c$ .

According to the IRT framework, the conditional response probabilities can be parametrized as follows:

(2) $$g[P\left(Y_{\mathrm{ik}}=r|\theta_{i},X_{i}=c\right)]=\alpha_{\mathrm{kc}}\left(\theta_{i}-\tau_{\mathrm{krc}}\right),\;r=1,\ldots ,m_{k},$$

where g(·) is the link function accounting for the binary or ordinal scoring of the considered indicators, $\alpha_{\mathrm{kc}}$ denotes the discrimination parameter of item k in latent class c , and $\tau_{\mathrm{krc}}$ represents the class-specific item-step difficulty parameters.

The individual posterior class membership probabilities $P\left(X_{i}=c|Y_{i}\right)$ can be calculated using Bayes’ theorem:

(3) $$P\left(X_{i}=c|Y_{i}\right)=\frac{\pi_{c}P\left(Y_{i}|X_{i}=c\right)}{\sum_{s=1}^{C}\pi_{s}P\left(Y_{i}|X_{i}=s\right)}.$$

When a vector of p individual covariates $Z_{i}=\left(Z_{i1},\ldots ,Z_{\mathrm{ip}}\right)$ is included in the model, they may affect both the structural component, influencing latent class membership, and the measurement component, directly affecting item response probabilities and inducing DIF.

The effect of covariates on latent class membership is specified through a multinomial logistic regression model:

(4) $$P\left(X_{i}=c|Z_{i}\right)=\frac{\exp\left(\gamma_{0c}+\gamma_{c}Z_{i}^{\prime }\right)}{\sum_{s=1}^{C}\exp\left(\gamma_{0s}+\gamma_{s}Z_{i}^{\prime }\right)},\;\;c=1,\ldots ,C,$$

where $\gamma_{0c}$ is the class-specific intercept and $\gamma_{c}$ is the vector of regression coefficients for covariates $Z_{i}$ on class c . For identifiability, one class is used as reference, usually $\left(\gamma_{01},\gamma_{1}=0\right)$ .

Conversely, covariates enter the measurement model as follows:

(5) $$g\left[P\left(Y_{\mathrm{ik}}=r|\theta_{i},X_{i}=c,Z_{i}\right)\right]=\alpha_{\mathrm{kc}}\left(\theta_{i}-\tau_{\mathrm{krc}}\right)\;+\;\beta_{\mathrm{krc}}Z_{i}^{\prime },\;\;r=1,\ldots ,m_{k},$$

where $\beta_{\mathrm{krc}}$ represents class-specific covariate effect (class-dependent DIF), which reduces to $\beta_{\mathrm{kr}}$ when the effect is invariant across latent classes (class-independent DIF).

### Estimation Procedure: The Proposed Two-Step Method

In LC-IRT models, parameter estimation can be carried out using either maximum likelihood (ML) or Bayesian approaches (De Ayala & Santiago, 2017). Among them, we adopt the marginal ML framework (Embretson & Reise, 2013), where the estimated parameters include class proportions $\pi_{c}$ , class-specific item parameters ( $\alpha_{\mathrm{kc}}$ , $\tau_{\mathrm{krc}}$ ), the population parameters of the class-specific continuous latent variables ( $\mu_{c}$ , $\sigma_{c}^{2}$ ), and, when included, regression coefficients for covariates ( $\gamma_{c}$ , $\beta_{\mathrm{krc}}$ ).

Let ϑ denote the set of model parameters; the full log-likelihood to be estimated is:

(6) $$\ell \left(\vartheta \right)=\sum_{i=1}^{n}\log P\left(Y_{i}|Z_{i}\right),$$

where

(7) $$P\left(Y_{i}|Z_{i}\right)=\sum_{c=1}^{C}P\left(X_{i}=c|Z_{i}\right)\int_{\Theta_{c}}\left[\overset{K}{\underset{k=1}{\Pi }}P\left(Y_{\mathrm{ik}}|\theta_{i},X_{i}=c,Z_{i}\right)\right]f_{c}\left(\theta_{i}\right)\;d\theta_{i}.$$

One-step estimation of the parameters can be obtained using software designed for latent variable modeling, in particular Latent Gold and Mplus, that typically use the EM algorithm, a quasi-Newton method, or a combination of them, to maximize the log likelihood in Equation 6.

The proposed two-step approach breaks down the estimation procedure into two steps. In the first step, the measurement model is estimated. When no DIF effects are included, this step is based only on the item responses and estimates the measurement model parameters $\vartheta_{1}=\left(\pi_{c},\alpha_{\mathrm{kc}},\tau_{\mathrm{krc}},\mu_{c},\sigma_{c}^{2}\right)$ . The corresponding Step 1 log-likelihood is:

(8) $$\ell_{1}\left(\vartheta_{1}\right)=\sum_{i=1}^{n}\log P\left(Y_{i}\right),$$

where $P\left(Y_{i}\right)$ is expressed according to Equation 1. When DIF effects are included in the final measurement model, let $Z_{i}^{\mathrm{DIF}}$ denote the subset of covariates that directly affect the item response probabilities. In this case, the corresponding direct effects on item response probabilities, $\beta_{\mathrm{krc}}^{\mathrm{DIF}}$ , are estimated in Step 1 as part of the measurement model. Following the recommendations of Vermunt and Magidson (2021), the DIF-inducing covariates are also included as predictors of latent class membership in Step 1. Thus, the first-step model includes the measurement parameters $\vartheta_{1}=\left(\pi_{c},\alpha_{\mathrm{kc}},\tau_{\mathrm{krc}},\mu_{c},\sigma_{c}^{2},\beta_{\mathrm{krc}}^{\mathrm{DIF}}\right)$ , together with the first-step regression coefficients $\gamma_{c}^{\mathrm{DIF}}$ , and the Step 1 log-likelihood becomes:

(9) $$\ell_{1}\left(\vartheta_{1},\gamma_{c}^{\mathrm{DIF}}\right)=\sum_{i=1}^{n}\log P\left(Y_{i}|Z_{i}^{\mathrm{DIF}}\right),$$

where

(10) $$P\left(Y_{i}|Z_{i}^{\mathrm{DIF}}\right)=\sum_{c=1}^{C}P\left(X_{i}=c|Z_{i}^{\mathrm{DIF}}\right)\int_{\Theta_{c}}\left[\overset{K}{\underset{k=1}{\Pi }}P\left(Y_{\mathrm{ik}}|\theta_{i},X_{i}=c,Z_{i}^{\mathrm{DIF}}\right)\right]f_{c}\left(\theta_{i}\right)\;d\theta_{i}.$$

In the second step, the measurement parameter estimates ${\tilde{\vartheta }}_{1}$ obtained in Step 1 are treated as fixed, and the structural model parameters $\vartheta_{2}=\left(\pi_{c},\gamma_{c}\right)$ are estimated, where $\gamma_{c}$ denotes the covariate effects on latent class membership. When DIF is included, the direct item-level DIF effects, $\beta_{\mathrm{krc}}^{\mathrm{DIF}}$ , are kept fixed from Step 1, while the covariate effects on latent class membership $\gamma_{c}^{\mathrm{DIF}}$ are re-estimated in the second-step structural model, together with any additional covariates of substantive interest to obtain the partial regression effects.

The log-likelihood for step 2 is $\ell_{2}\left({\tilde{\vartheta }}_{1},\vartheta_{2}\right)$ , which is equal to $\sum_{i=1}^{n}\log P\left(Y_{i}|Z_{i}\right)$ , with $P\left(Y_{i}|Z_{i}\right)$ defined in the most general form as in Equation (7), evaluated at the Step 1 estimates of the measurement parameters ${\tilde{\vartheta }}_{1}$ , excluding the covariate effects on latent class membership $\gamma_{c}^{\mathrm{DIF}}$ , which are included in $\vartheta_{2}$ and re-estimated in Step 2. $\ell_{2}\left({\tilde{\vartheta }}_{1},\vartheta_{2}\right)$ is maximized with respect to $\vartheta_{2}$ to obtain the two-step estimate of structural model parameters.

The standard error estimates of the Step 1 and step 2 models can be obtained using the standard Hessian-based estimators available in all software packages. For the second step model these estimates are known to be downward biased, due to ignoring the variability in the fixed parameters (Bakk & Kuha, 2018). However, this attenuation bias is known to be small in complex models (Di Mari & Bakk, 2018), while the correction is complex enough to be ignored in practice—as such we test in the simulation experiment the bias encountered using the standard estimator.

The two-step estimation approach provides both methodological and practical advantages by separating the measurement and structural components of the model (Bakk & Kuha, 2018). This separation allows the structural model to be modified without re-estimating the measurement model, ensures computational efficiency, and facilitates model comparison. Interpretability is enhanced by the two-step approach since the measurement model is estimated separately from the structural covariate model, preserving the substantive meaning of latent classes. In addition, it enables the use of different samples across estimation stages, which is particularly valuable when auxiliary variables are available only for subsamples. These properties make the two-step approach particularly useful for LC-IRT models.

### Multidimensional Latent Class IRT Models and Two-Step Estimation

The use of two-step estimation can be naturally extended to more complex situations, such as multidimensional latent traits in LC-IRT models (e.g., see Bacci et al., 2017, 2014). Suppose that we have more than one dimension of the latent trait $\theta_{i}=\left(\theta_{i1},\ldots ,\theta_{\mathrm{iD}}\right)$ , measured by separate sets of indicators (between-item multidimensionality), producing the full response vector $Y_{i}$ for respondent i . Simultaneously, as typical in LC-IRT models, a discrete latent categorical variable $X_{i}\in \{1,\ldots ,C\}$ indexes latent classes that partition the population into distinct subgroups. Each latent class c is characterized by a class-specific distribution of the continuous latent traits, typically modeled as a multivariate normal distribution with mean vector $\mu_{c}$ and covariance matrix $\Sigma_{c}$ . As in the unidimensional case, a vector of p covariates $Z_{i}$ may enter the model in the structural component, predicting latent class membership probabilities through the multinomial logistic regression model defined in Equation (4), or in the measurement component, directly influencing item response probabilities and thereby inducing DIF. The most general specification allows covariates to operate on both components simultaneously.

In the multidimensional setting, the marginal response probability in Equation (7) is naturally generalized to the multivariate latent trait case, and, when covariates are allowed to directly affect the indicators, the conditional response probabilities in Equation (2) become:

(11) $$g[P\left(Y_{\mathrm{ik}}=r|\theta_{i},X_{i}=c,Z_{i}\right)]=\alpha_{\mathrm{kc}}\left(\sum_{d=1}^{D}I_{\mathrm{kd}}\theta_{\mathrm{id}}-\tau_{\mathrm{krc}}\right)+\beta_{\mathrm{krc}}Z_{i}^{\prime },\;r=1,\ldots ,m_{k},$$

where g(·) is the link function accounting for the binary or ordinal scoring of the considered indicators, $\alpha_{\mathrm{kc}}$ denotes the discrimination parameter of item k in latent class c , $\tau_{\mathrm{krc}}$ represents the class-specific item-step difficulty parameters, and $I_{\mathrm{kd}}$ is a dummy variable equal to 1 if the item k measures the latent trait d . The term $\beta_{\mathrm{krc}}$ denotes the class-specific covariate effect on item k (class-dependent DIF), which reduces to $\beta_{\mathrm{kr}}$ when the effect is invariant across latent classes (class-independent DIF).

Under the proposed two-step approach, the estimation procedure follows the same rationale as in the unidimensional case. In the first step, the multidimensional measurement model is estimated. When no DIF effects are specified, this step is based on the item responses only and involves estimating the class proportions $\pi_{c}$ , class-specific item parameters, and the parameters of the latent trait distributions $\left(\mu_{c},\Sigma_{c}\right)$ for each class. When DIF effects are included in the final measurement model, the first-step model is extended to condition the item response probabilities on the DIF-inducing covariates, $Z_{i}^{\mathrm{DIF}}$ . In this case, the direct covariate effects on item response probabilities, $\beta_{\mathrm{krc}}^{\mathrm{DIF}}$ , and the effects of $Z_{i}^{\mathrm{DIF}}$ on latent class membership, $\gamma_{c}^{\mathrm{DIF}}$ , are estimated in Step 1 together with the other measurement parameters.

In the second step, the measurement parameter estimates ${\tilde{\vartheta }}_{1}$ obtained in Step 1 are treated as fixed, and covariates are used to estimate their effects on latent class membership probabilities, leading to the estimation of the structural parameters $\vartheta_{2}=\left(\pi_{c},\gamma_{c}\right)$ . When DIF is present, the direct effects of covariates on item responses, $\beta_{\mathrm{krc}}^{\mathrm{DIF}}$ , are kept fixed, whereas the regression coefficients of latent class membership on the DIF-inducing covariates, $\gamma_{c}^{\mathrm{DIF}}$ , are re-estimated in the Step 2 structural model, together with any additional covariates of substantive interest, following the recommendations of Vermunt and Magidson (2021).

The corresponding step 1 and step 2 log-likelihood functions, $\ell_{1}\left(\vartheta_{1},\gamma_{c}^{\mathrm{DIF}}\right)$ and $\ell_{2}\left({\tilde{\vartheta }}_{1},\vartheta_{2}\right)$ , retain the same structure as in the unidimensional case, with the latent trait distribution and response model generalized to the multidimensional case. By separating the estimation of the measurement and structural components, this approach can reduce computational complexity and mitigate convergence issues that may arise in multidimensional models. This step-wise procedure is particularly advantageous in multidimensional LC-IRT settings, where joint estimation of measurement and structural components is often computationally demanding and prone to convergence problems.

## Simulation Experiment

We performed an extended simulation experiment to investigate the performance of the simultaneous and the proposed two-step estimator for complex LC-IRT models. Namely, we conducted a simulation study that investigates the performance of the two estimators for LC-IRT models defined by a single continuous latent variable under the 1PL model with binary items (Study 1), a study with a 2PL model and ordinal items (Study 2) and a study with a multidimensional model, where the latent classes are defined by the combination of three continuous latent variables, a setup motivated by our real data application (Study 3). In all three studies, data was generated under models with and without DIF and analyzed using the correct and misspecified models.

### Study 1: Latent Classes Defined by a Single Factor in the Presence of DIF

In the first simulation experiment, we compared the performance of the simultaneous and two-step estimators under models where data was generated from a single latent factor. We used the simplest LC-IRT model, namely the 1PL Rasch model. The reason for this choice is that this model is still the most popular, most often used LC-IRT model: according to Noroozi and Karami (2026), it is used in 42% of the articles included in their extensive literature review. The goal of the simulation study was to compare the performance of the two estimators under three scenarios: a simple model, where no DIF of covariates on indicators exists (meeting the main assumption of conditional independence), and two scenarios where data are generated under violations of the conditional independence assumption, namely class-independent DIF and the more complex class-specific DIF.

Previous simulation studies focusing on the stepwise estimation of simpler latent class models show that the performance of the different estimators with respect to bias and efficiency depends on class separation and class imbalance (Asparouhov & Muthén, 2014b; Bakk & Kuha, 2018; Di Mari & Bakk, 2018; Masyn, 2017; Vermunt, 2010). We manipulated class separation by changing the number of items, namely 5, 10, and 30 items measuring the latent factor, and the sample sizes, namely 500 and 2,000. We generated data from a model with two latent classes, under two conditions of class sizes, namely equal class sizes, and unbalanced class sizes (0.30 and 0.70) for all models. Two continuously distributed covariates were added, under two scenarios: large effect of the covariate on the latent classes (LCs), and small effects. Specifically, the logit coefficient of $Z_{1}$ on the latent classes in the high effect size condition was set to 2.079, while in the low effect size condition to 0.405. For $Z_{2}$ the high effect size was generated from a logit coefficient of −1.387, and in the low effect size condition of −0.205. Furthermore, in all conditions, we ran 100 replications. The items were generated to measure the latent factors using a 1PL model with class-specific intercepts with a medium to large effect size (1.345 in class 1, and −1.345 in class 2) with fixed factor loadings (1). In class 1 all items have a high intercept, while in class 2 a low intercept, corresponding to a high and low competence class.

**Figure 1.**
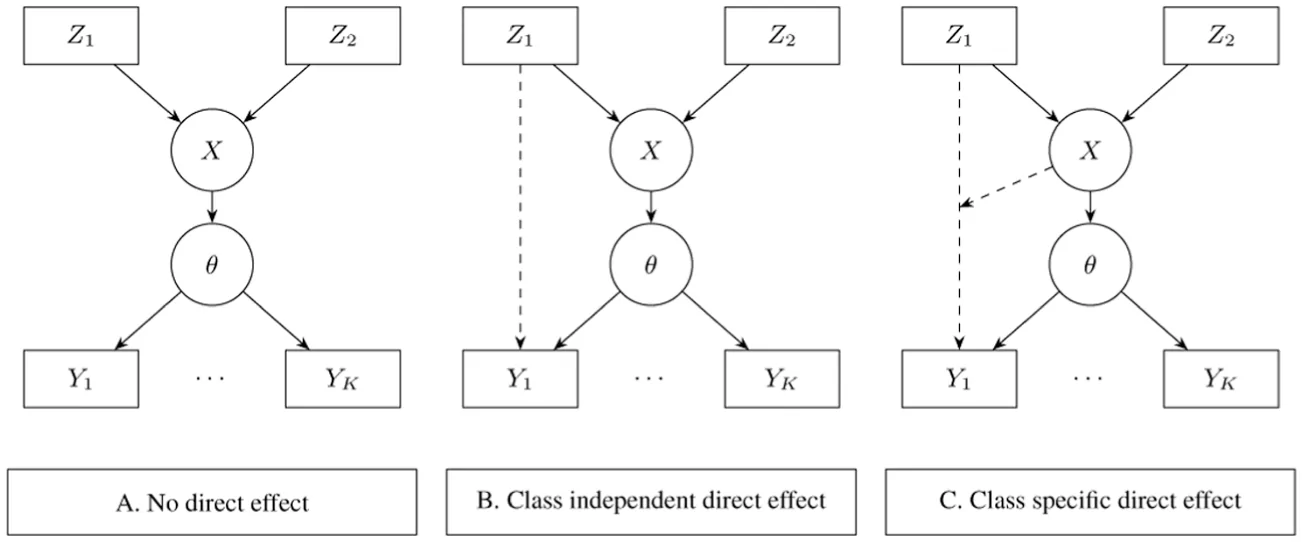
Population models for the simulation experiment of Study 1.

Data was generated from three population models, namely a model where the conditional independence assumption of the items from the covariates is met (graph A in Figure 1), and two models where this assumption is violated, namely the simpler class-independent DIF (graph B in Figure 1) and the most complex class specific DIF (graph C in Figure 1). Under population model B, a DIF of $Z_{1}$ on item 2 was generated with an effect size of 0.693—this means that item 2 becomes uninformative, as class 1 and class 2 score very close to each other on item 2. Under model C, we generated data with DIF on item 2 as well, the DIF was set to 0.43 in both classes—as such the effect size of the DIF is smaller than under model B, and the score on item 2 is closer to each other between the 2 classes, but item 2 still differentiates between the classes.

Overall, under each of the three data generating models, three setups for number of indicators, two class sizes, two sample sizes scenarios were considered. Under all data generating conditions, four data analyzing models were applied, namely the three data generating models, and a model where the class-independent DIF of $Z_{2}$ is modeled on an indicator (item 2), to mimic a scenario when a wrong DIF is identified.

In all simulation conditions, we investigate the performance of the estimators with regard to bias (defined as the difference between the parameter estimate and the population value) and the 95% coverage rate (percentage of samples where the true value lies within the 95% confidence interval). We also investigate the effect of entropy for the one step model, and for the step one model for the two-step estimator, as previous simulation studies of LC models have shown that the stepwise estimators tend to be biased when the entropy is low. The item bias of the measurement model is also reported, together with the DIF parameters and Type 1 error rate for the wrong models where available.

### Results Study 1

#### Data Generating Model A: No DIF

**Figure 2.**
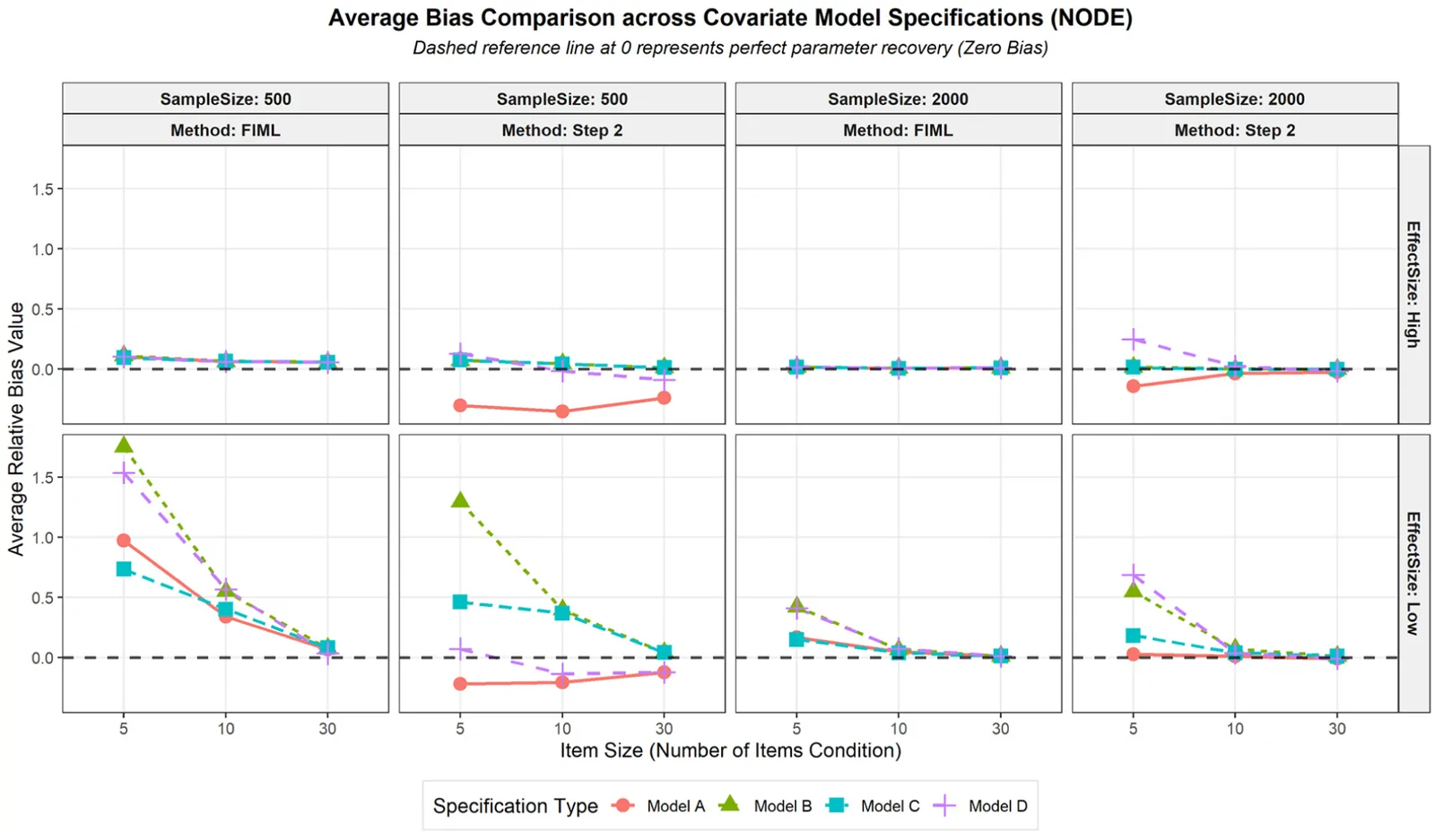
Bias in the structural parameters for FIML and the two-step estimator under the different simulation scenarios under the data-generating condition Model A: NO DIF. *Note*. DIF = differential item functioning; FIML = full information maximum likelihood.

Figure 2 shows the bias in the structural parameters under data generating condition Model A. In the low effect size condition, the bias is larger with both estimators, and the two-step estimator is more biased in conditions with a weaker measurement model (conditions with five items). In conditions where the sample size is large, and the number of items is also large, both estimators are essentially unbiased.

**Figure 3.**
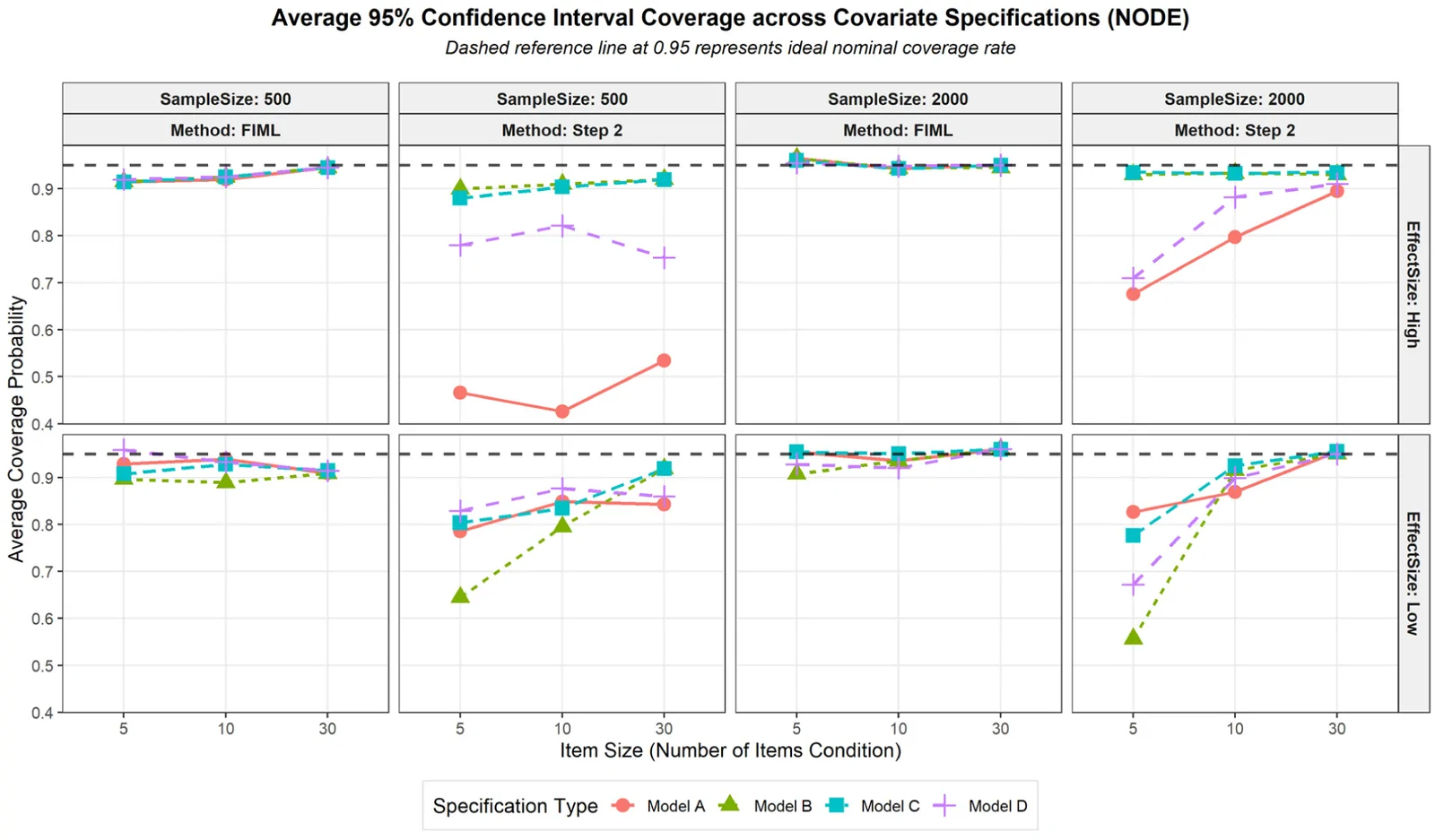
Coverage of the structural parameters for FIML and the two-step estimator under the different simulation scenarios under the data-generating condition Model A: NO DIF. *Note*. DIF = differential item functioning; FIML = full information maximum likelihood.

Furthermore, Figure 3 shows the coverage for these parameters, taking into account also the information on standard errors. This graph clearly shows that using the two-step estimator ignoring the variance due to the fixed parameters from step 1 introduces a bias in the standard errors, causing a larger variability in coverage for this approach.

**Figure 4.**
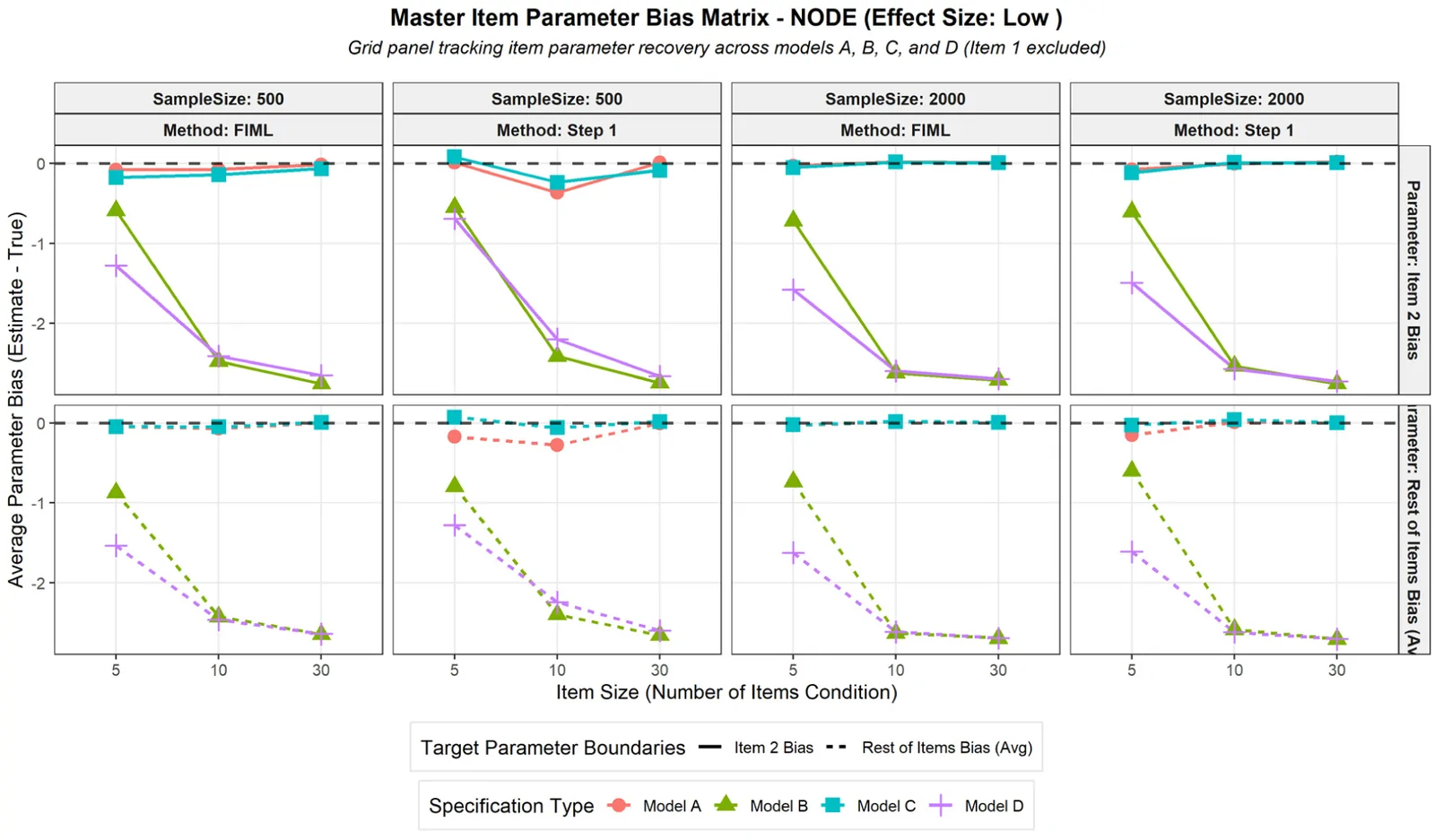
Average item bias for FIML and the two-step estimator under the different simulation scenarios under the data generating condition Model A: NO DIF. *Note*. DIF = differential item functioning; FIML = full information maximum likelihood.

At the same time, Figure 4 shows the bias in item parameters. Data analysis models B and D, which assume a DE, are clearly more biased than model A. Interestingly, model C, which assumes a more complex class-specific DIF is less biased than models B and D. Note that models B, C, and D model a DIF on item 2.

To better understand the results, we ran a regression analysis for predicting average bias in the structural parameters. The regression results in Table 1 demonstrate that the proposed stepwise approach provides a reliable framework for structural estimation, though its performance is conditioned by measurement model quality. Entropy emerged as a significant negative predictor of bias, confirming that higher measurement precision directly enhances structural recovery.

**Table 1.** Regression Analysis of Average Structural Bias Under Data Generating Model A.

Predictor	Estimate	Std. error	*p*-Value
(Intercept)	0.622	0.207	.003
Method (FIML)	0.522	0.101	<.001
Model (B)	0.418	0.089	<.001
Model (C)	0.357	0.091	<.001
Model (D)	0.433	0.079	<.001
Class size (equal)	0.033	0.039	.409
Sample size (2,000)	−0.124	0.039	.002
Effect size (low)	0.048	0.071	.502
Item size (10)	−0.158	0.055	.005
Item size (30)	−0.136	0.077	.078
Entropy	−1.256	0.375	.001
Method (FIML) × Model (B)	−0.328	0.118	.006
Method (FIML) × Model (C)	−0.361	0.119	.003
Method (FIML) × Model (D)	−0.352	0.111	.002
$R^{2}$	.466	—	—
Adj. $R^{2}$	.427	—	—

*Note*. FIML = full information maximum likelihood.

FIML showed higher average bias than the stepwise estimator under the reference specification, although the negative interaction terms indicate that this difference decreased under Models B, C, and D. These findings suggest that the stepwise estimator can provide a viable alternative to FIML when the measurement model is sufficiently strong.

#### Data Generating Model C: Class Specific DIF

The results for data generated under model C and model B are very similar; as such, we only present the more complex model C. Detailed simulation results for data generated under model B are available in the Supplemental Material available on OSF.^1^

**Figure 5.**
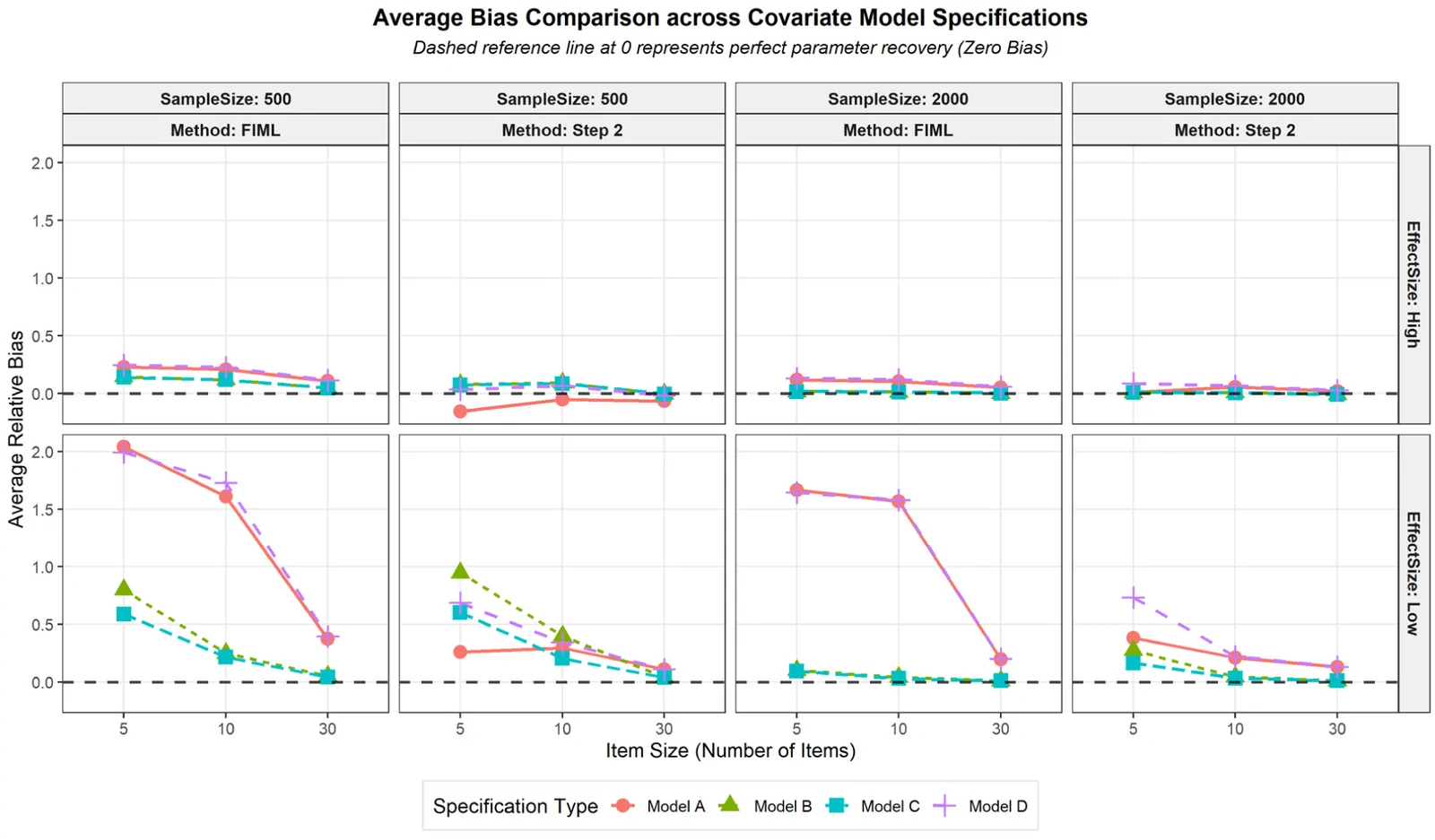
Bias in the structural parameters for FIML and the two-step estimator under the different simulation scenarios under the data-generating condition Model C: Class-specific DIF. *Note*. DIF = differential item functioning; FIML = full information maximum likelihood.

**Figure 6.**
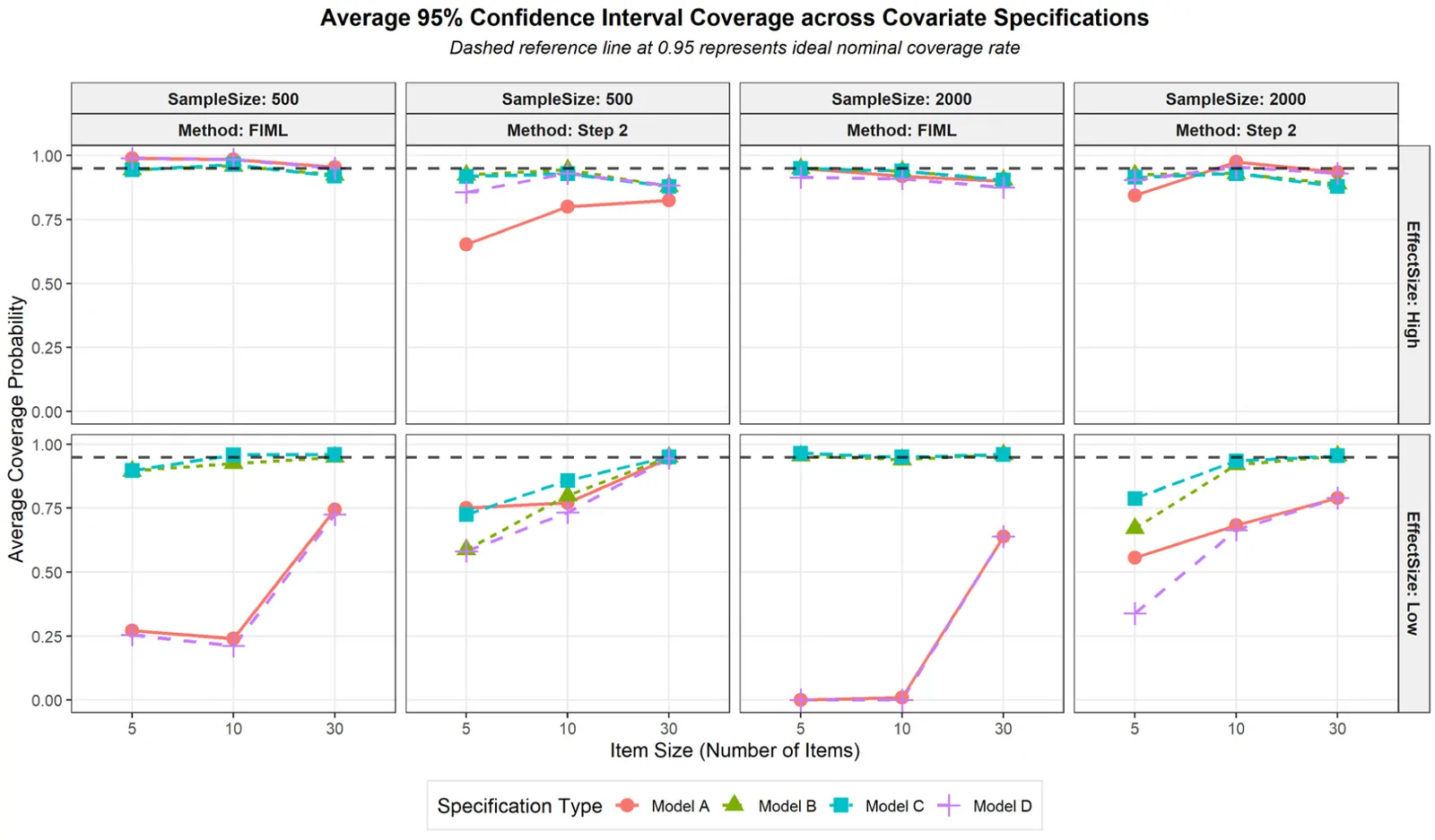
Coverage of the structural parameters for FIML and the two-step estimator under the different simulation scenarios under the data-generating condition Model C: Class-specific DIF. *Note*. DIF = differential item functioning; FIML = full information maximum likelihood.

Figures 5 and 6 present the bias and coverage of the structural parameters. FIML shows a higher sensitivity to model misspecification in this complex setup than the stepwise estimator.

**Figure 7.**
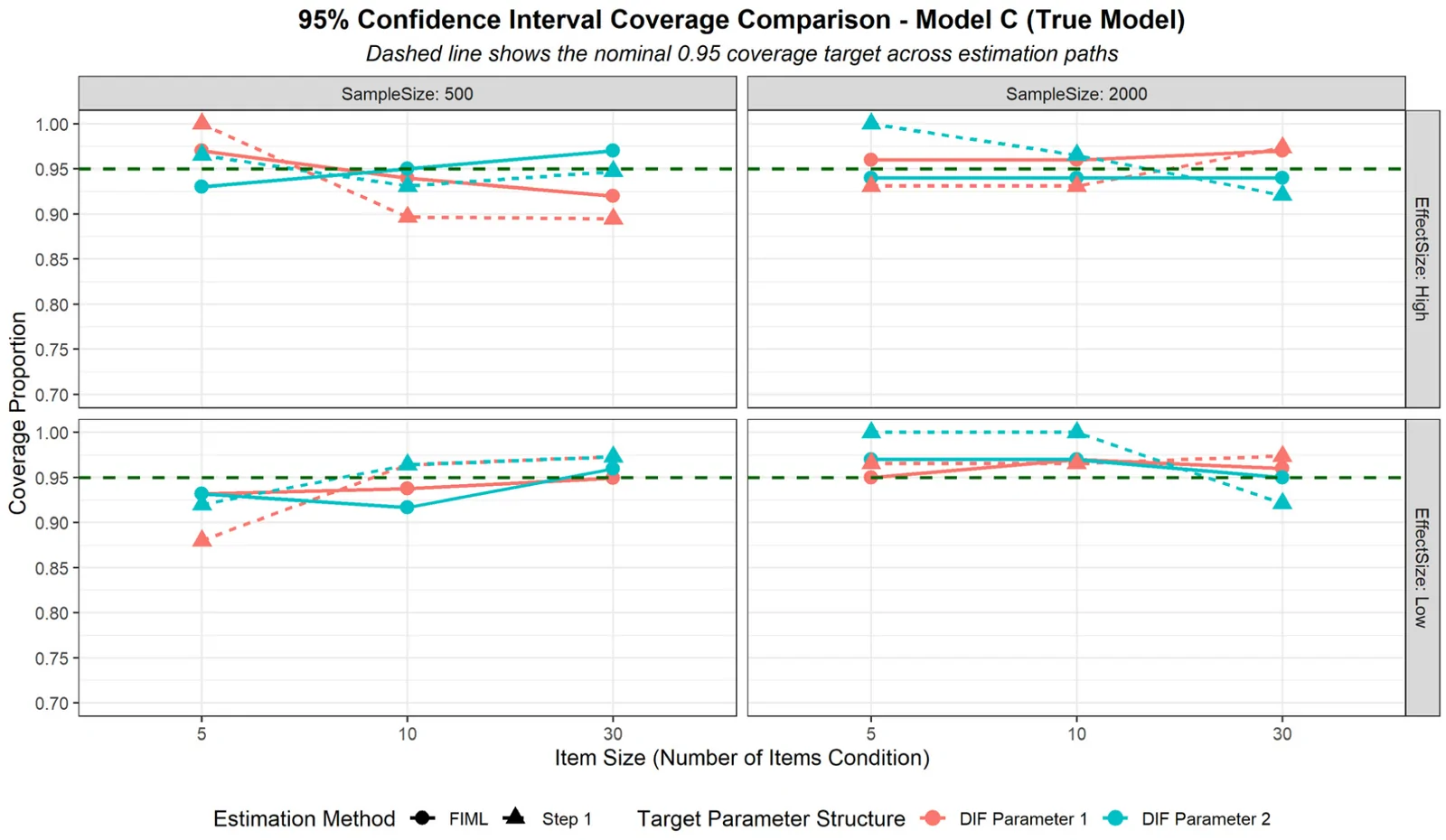
Coverage of the DIF parameters for FIML and the two-step estimator under the different simulation scenarios for the data-generating Model C: Class-specific DIF. *Note*. DIF = differential item functioning; FIML = full information maximum likelihood.

The parameters of the direct effect are recovered well with both estimators under most conditions, as we can see in Figure 7.

**Figure 8.**
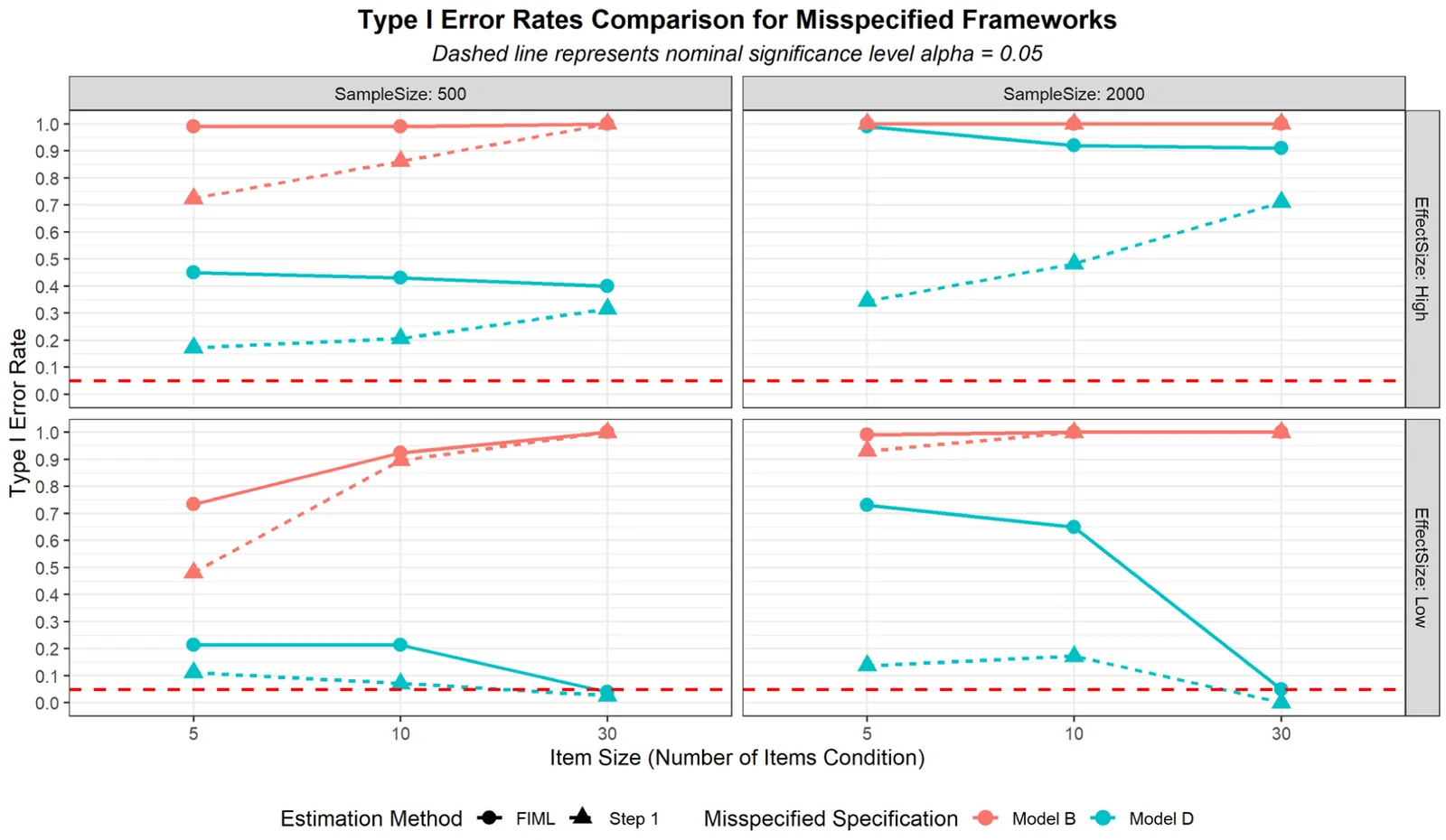
Type I error rates for the incorrectly specified DIF effects under data-analysis Models B and D, for FIML and the two-step estimator across simulation scenarios under the data-generating Model C (class-specific DIF). *Note*. DIF = differential item functioning; FIML = full information maximum likelihood.

Type I error rates for misspecified DIF effects were often above the nominal level, as we can see in Figure 8, particularly for Model B and for some Model D conditions.

**Figure 9.**
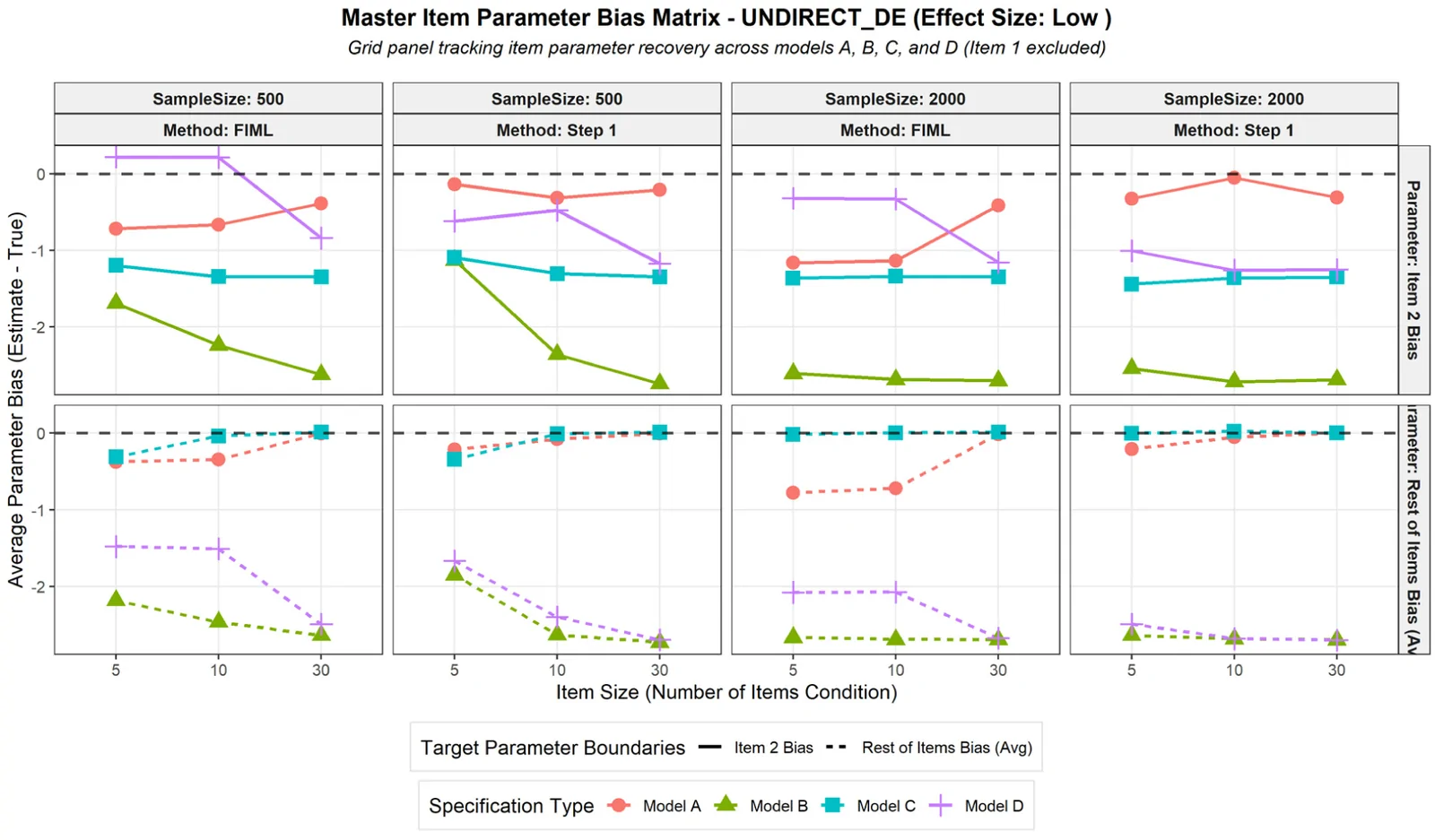
Average item bias for FIML and the two-step estimator under the different simulation scenarios under the data-generating condition Model C. DIF in Item 2. *Note*. DIF = differential item functioning; FIML = full information maximum likelihood.

At the same time, Figure 9 shows the bias in item parameters in the low effect size condition. The models that assume a wrong DIF have the highest bias, even in the items that are not affected by DIF, so there is an overflow effect. In the large effect size condition, results with regard to item bias are comparable, and available in the Supplemental Material available on OSF.

Under the condition where Model C is the true data-generating mechanism, the results show a robust model fit ( $R^{2}=.689$ ) for the regression model on average bias of the structural parameters (Table 2). Entropy remains the strongest negative predictor of bias, reinforcing the conclusion that measurement quality is essential for structural parameter recovery.

**Table 2.** Regression Analysis of Average Structural Bias Under Data Generating Model C.

Predictor	Estimate	Std. error	*p*-Value
(Intercept)	1.150	0.155	<.001
Method (FIML)	0.596	0.064	<.001
Model (B)	0.219	0.061	<.001
Model (C: True)	0.201	0.061	.001
Model (D)	0.240	0.058	<.001
Class size (Equal)	0.008	0.029	.778
Sample size (2,000)	−0.126	0.029	<.001
Effect size (Low)	−0.039	0.052	.457
Item size (10)	0.058	0.040	.153
Item size (30)	0.090	0.059	.131
Entropy	−2.005	0.261	<.001
Method (FIML) × Model (B)	−0.443	0.082	<.001
Method (FIML) × Model (C)	−0.436	0.082	<.001
Method (FIML) × Model (D)	−0.058	0.081	.478
$R^{2}$	.689	—	—
Adj. $R^{2}$	.666	—	—

*Note*. FIML = full information maximum likelihood.

While the FIML estimator shows higher average bias than the two-step approach, the significant negative interaction terms for Models B and C indicate that FIML’s performance gap narrows considerably in these configurations. Overall, the two-step approach maintains a distinct advantage in computational stability and bias reduction, particularly when entropy is large enough in the step one model.

Furthermore, for completeness, we should mention that the valid sample size fluctuated in the conditions with five items, while it was consistently high (100, or very close to it) in the other conditions. The two-step estimator had a smaller converged sample size in the five item, 500 sample size conditions. In the most complex Model C simulation study, the convergence rate was between 0.73 and 0.90 and the entropy between 0.38 and 0.48. For FIML in this condition, the convergence rate was between 0.87 and 1 and the entropy between 0.46 and 0.53. In the other conditions, results are comparable.

### Study 2: LC-IRT Model With Ordinal Items and Three Classes

To evaluate the robustness of the structural estimation across different measurement conditions, we conducted a simulation study using an ordinal LC-IRT model with three latent classes. The population was generated with three latent classes of unequal sizes, defined by proportions of 0.27, 0.365, and 0.365 for classes 1, 2, and 3, respectively. The structural model included two continuous covariates, $Z_{1}$ and $Z_{2}$ . Effect sizes for $Z_{1}$ coefficients were 0.15 (Class 2) and −0.15 (Class 3), and the $Z_{2}$ coefficients were 0.60 (Class 2) and -0.60 (Class 3). Two sample size conditions, namely 500 and 2,000, were considered. The measurement model consisted of 10 ordinal items, each with three categories. For each of the 10 items, two category thresholds and a constant discrimination parameter of 1.2 across all latent classes were used. The threshold structures were systematically varied to distinguish between the three latent classes: items $Y_{1}$ through $Y_{3}$ follow a (1,−1),(−1,1),(1,−1) pattern; items $Y_{4}$ and $Y_{5}$ follow a (1,−1),(−1,1),(−1,1) pattern; and items $Y_{6}$ through $Y_{10}$ mirror the structure of $Y_{4}$ and $Y_{5}$ with a (1,−1),(−1,1),(−1,1) configuration. In the DIF condition, a 0.5 threshold shift was introduced on Item 1 to simulate DIF between classes. In each condition, 100 replications were analyzed using FIML and two-step estimator, modeling and ignoring DIF (so a wrong and a correct model per condition of DIF). The variance of the latent factor was set to 1.

### Results Study 2

The key results for the structural parameters are reported in Table 3. Even though the entropy is higher for the FIML methods, we can see that the convergence rate (namely the number of samples that converged out of 100 replications) is lower with FIML for these more complex models. Both estimators have very low bias, and the coverage is consistently better with FIML; that is due to the standard error underestimation with the stepwise estimator.

**Table 3.** Simulation Results: Mean Bias and Coverage for the Structural Parameters by Method, Sample Size, and Condition for Study 2.

Method	*N*	Model	Converged	Entropy	M. bias Z1	M. Cov. Z1	M. bias Z2	M. Cov. Z2
Data generated under no DIF								
2-Step	500	Correct	93	0.71	0.01	0.96	0.03	0.88
2-Step	500	Wrong	89	0.71	0.01	0.94	0.04	0.86
2-Step	2,000	Correct	91	0.68	0.00	0.93	0.02	0.90
2-Step	2,000	Wrong	97	0.69	0.00	0.93	0.02	0.91
FIML	500	Correct	51	0.75	−0.01	0.96	0.02	0.95
FIML	500	Wrong	51	0.75	−0.03	0.96	0.01	0.95
FIML	2,000	Correct	62	0.73	0.00	0.97	0.02	0.94
FIML	2,000	Wrong	67	0.73	0.00	0.97	0.03	0.96
Data generated under DIF								
2-Step	500	Wrong	85	0.70	0.01	0.88	0.03	0.94
2-Step	500	Correct	88	0.70	0.00	0.89	0.02	0.94
2-Step	2,000	Wrong	94	0.67	0.01	0.91	0.01	0.93
2-Step	2,000	Correct	95	0.68	0.01	0.94	0.01	0.95
FIML	500	Wrong	37	0.74	−0.01	0.97	−0.01	1.00
FIML	500	Correct	43	0.76	−0.01	0.99	−0.02	0.97
FIML	2,000	Wrong	40	0.73	0.00	0.96	0.00	1.00
FIML	2,000	Correct	39	0.73	−0.01	0.99	0.00	0.97

*Note*. DIF = differential item functioning; FIML = full information maximum likelihood.

Table 4 reports the bias of item 1 (for the rest of the items, the bias is similar) for both the discrimination and threshold parameters. For all models, we can see that the bias is negligible. Also, the Type 1 error rate for the wrong models is small. Furthermore, Table 5 reports the item bias for the same item, and also the bias of the DIF parameter for the models where this was estimated. The results also under this condition show small bias for the DIF parameter and the threshold, however for the models that ignore the DIF, with both estimators we can see that the discrimination parameter is biased. Furthermore, with regard to model stability we can report that for these more complex models the FIML approach had more datasets with convergence problems (convergence rate between 37% and 67%), while the step one models had a better convergence rate (above 85%).

**Table 4.** Average Bias for $Y_{1}$ in Discrimination and Threshold Parameters for Data Generated Under No DIF Condition.

Method	*N*	Bias (discrim.)	Bias (thresh)	Type I error
FIML wrong	500	−0.0496	0.0584	0.04
FIML wrong	2,000	−0.0142	0.0037	0.08
FIML correct	500	−0.0114	0.0725	—
FIML correct	2,000	−0.0177	0.0018	—
Step one wrong	500	−0.0647	0.0980	0.01
Step one wrong	2,000	−0.0108	0.0231	0.01
Step one correct	500	0.0463	0.0986	—
Step one correct	2,000	0.0186	0.0171	—

*Note*. DIF = differential item functioning; FIML = full information maximum likelihood.

**Table 5.** Average Bias for $Y_{1}$ in Discrimination and Threshold Parameters for Data Generated Under DIF Condition.

Method	*N*	Bias (discrim.)	Bias (thresh)	Bias DIF
FIML correct	500	−0.0302	0.0369	0.0109
FIML correct	2,000	−0.0294	0.0276	0.0031
FIML wrong	500	1.5140	−0.0736	—
FIML wrong	2,000	1.3594	−0.0746	—
Step one correct	500	−0.0584	0.1572	0.0334
Step one correct	2,000	−0.0138	0.0184	−0.0008
Step one wrong	500	1.5131	0.0530	—
Step one wrong	2,000	1.3635	−0.0649	—

*Note*. DIF = differential item functioning; FIML = full information maximum likelihood.

### Study 3: Multidimensional LC-IRT

In Study 3 we investigated the performance of the FIML and stepwise estimator in a multidimensional LC-IRT model, where data was generated from a model with three factors (each with a variance of 1, and no covariance), each factor is measured by five binary items using a 1PL model. A two-latent-class model was employed, the class membership being predicted by three covariates, $Z_{1}$ (no effect), $Z_{2}$ (large effect, 1.387), and $Z_{3}$ (low effect, −0.205). The intercepts of class sizes were set so that class 1 has a size of 0.30, and class 2 of 0.70. Items $Y_{1}$ to $Y_{10}$ , that load on Factor 1 ( $Y_{1}-Y_{5}$ ) and Factor 2 ( $Y_{6}-Y_{10}$ ) are set to have a threshold of 1.345 in class 1 and −1.345 in class 2. Items $Y_{11}-Y_{15}$ loading on Factor 3 have the opposite pattern. Data was generated under a no DIF and a DIF model. In the DIF model, items $Y_{1},Y_{11}$ have a DIF of 0.5, and item $Y_{6}$ of −0.5, creating a setup where each factor has DIF in the first item. Under the no DIF model we investigated the small sample performance of the estimators as well (we did not do this for the DIF models, given the very long estimation time: approximately 1 hr per model for the larger sample sizes). In the no DIF scenario, we had four sample size scenarios: 250, 500, 750, and 2,000, and only the correct model that assumes no DIF. In the DIF scenario, we had 2 sample size scenarios: 500, 2,000, and we estimated with both FIML and stepwise estimators models that assume or ignore DIF.

### Results Study 3

Table 6 presents the main results for the data generated under the no DIF condition. Columns 2 to 7 show Bias and coverage in the covariate effects. In all sample size conditions, the bias is low, and the coverage is close to the nominal rate for both estimators. Furthermore, the bias of the variance of the latent factors is also low for both estimators, and the coverage is also acceptable. The item bias is negligible under all conditions. The good performance of the estimators can probably be explained by the very high entropy values in the last column. There were no samples with convergence problems.

**Table 6.** Average Bias, Coverage and Entropy for Data Generated Under No DIF Conditions.

*N*	B1	Cov1	B2	Cov2	B3	Cov3	BVar	CovVar	B_item	Entropy
FIML										
250	0.006	0.96	0.081	0.94	0.013	0.97	−0.008	0.97	0.007	0.928
500	−0.011	0.95	0.008	0.96	−0.014	0.92	−0.004	0.96	0.003	0.908
750	0.005	0.95	0.005	0.96	−0.007	0.95	0.001	0.95	0.005	0.880
2,000	0.001	0.88	0.012	0.95	−0.004	0.99	0.003	0.95	0.000	0.891
Step two										
250	0.007	0.96	0.033	0.92	0.020	0.96	−0.013	0.94	0.014	0.878
500	−0.011	0.95	−0.008	0.95	−0.010	0.92	−0.005	0.95	0.049	0.890
750	0.005	0.96	−0.010	0.97	−0.007	0.95	0.000	0.93	0.051	0.836
2,000	0.001	0.88	0.008	0.96	−0.004	0.99	0.003	0.94	0.046	0.821

*Note*. DIF = differential item functioning; FIML = full information maximum likelihood.

Table 7 presents a summary of the primary findings for data generated under the DIF simulation conditions. Columns 3 through 8 report the bias and the coverage for the structural parameters; these results demonstrate negligible performance discrepancies between the FIML and two-step estimators. Columns 9 and 10 provide an evaluation of the bias and coverage ratio for the variance components of the three factors, showing similarly low bias and good coverage, with slight underestimation of the coverage for the stepwise estimator. Column 11 reports the average bias for the DIF parameters, indicating high recovery accuracy with minimal bias across both estimators.

**Table 7.** Average Bias, Coverage of the Structural Parameters, Bias in DIF and Item Parameters for Data Generated Under DIF Conditions.

Method	*N*	B1	Cov1	B2	Cov2	B3	Cov3	B_Var	Cov_Var	B_DIF	B_Items
DIF models											
FIML_DIF	500	0.005	0.93	0.024	0.97	0.004	0.94	−0.003	0.96	0.010	0.178
FIML_DIF	2,000	0.00	0.99	0.007	0.95	−0.001	0.94	0.006	0.95	0.007	0.181
Steptwo_DIF	500	0.004	0.93	0.006	0.96	0.007	0.94	−0.004	0.94	0.011	0.009
Steptwo_DIF	2,000	0.00	0.98	0.001	0.95	−0.001	0.94	0.004	0.94	0.006	0.006
Non-DIF models											
FIML	500	0.026	0.92	0.026	0.97	0.004	0.97	−0.018	0.95		0.037
FIML	2,000	0.022	0.949	0.008	0.95	0.000	0.949	−0.008	0.95		0.045
Steptwo	500	0.026	0.92	0.009	0.96	0.007	0.96	-0.019	0.93		0.098
Steptwo	2,000	0.022	0.95	0.004	0.95	0.001	0.95	−0.009	0.93		0.095

*Note*. DIF = differential item functioning; FIML = full information maximum likelihood.

Finally, the last column reports the average item bias. Overall, this bias is generally low for the two-step models, whereas FIML shows larger item bias in the DIF models.

The consistent low bias and good coverage across the methods can also be explained by a high average entropy, namely above .80 in all conditions for all models. There were no samples with convergence problems.^2^

## An Application to Real Data on Learning Statistics

In this section, we present an application of the two-step estimation approach to a real-data case study regarding learning statistics in non-STEM degree courses. Data were collected from a sample of n=300 Italian undergraduate students, enrolled in a psychology degree program, during the Statistics course in the academic years 2022 and 2023. Participants were predominantly female (75%) , and their age ranged from 18 to 47 years (mean = 19.95 , *SD* = 2.82 ).

Students’ ability in Statistics was conceived as multidimensional according to Dublin descriptors (Gudeva et al., 2012), which are general statements used to evaluate the knowledge depth a student has achieved within a specific topic at the end of each cycle of higher education studies. Specifically, we considered the following three Dublin descriptors: (a) knowledge and understanding, namely the ability to demonstrate knowledge and understanding, including a theoretical, practical and critical perspective on the topic (herein referred to as *Knowledge* dimension); (b) applying knowledge and understanding, namely the ability to apply the knowledge identifying, analyzing and solving problems sustaining an argument (herein, referred to as *Application* dimension); (c) making judgments, namely the ability to gather, evaluate and present information exercising appropriate judgment (herein, referred to as *Judgment* dimension). For each of the three dimensions, 10 multiple-choice questions were developed, resulting in a total of 30 indicators.^3^ Questions were administered through the Moodle platform, a learning management system commonly adopted in higher education for course delivery, assessment of students’ competencies, and provision of formative feedback, and covered topics such as descriptive statistics, graphs, tables, and Gaussian distribution. Responses were evaluated using a three-level scoring scheme: fully correct answers received two points, partially correct answers received one point, and incorrect answers received zero points. Missing responses were treated as missing data.

Several demographic and psychological characteristics (i.e., sex, math knowledge, academic motivation, self-efficacy, engagement in statistics) were also assessed at the beginning of the course or, in the case of engagement, a few days before the statistics test. The considered students’ characteristics have been shown in the literature to influence learning outcomes. Male students often outperform female students in STEM-related subjects (Cipora et al., 2015; Else-Quest et al., 2010). Prior mathematical knowledge represents a key prerequisite for Statistics learning, as severe math shortcomings could constitute a roadblock to completing statistics courses successfully (Johnson & Kuennen, 2006; Rabin et al., 2018). Motivational and affective components also play a crucial role in shaping students’ academic performance: lower levels of academic motivation have been associated with frustration and disengagement, which in turn negatively affect learning outcomes (Cokley et al., 2001; Steel et al., 2001); similarly, self-efficacy influences students’ confidence in dealing with statistical tasks and their persistence when facing difficulties, particularly in non-STEM contexts where Statistics is often perceived as challenging (Honicke & Broadbent, 2016; Richardson et al., 2012). Finally, higher levels of engagement in Statistics (e.g., active participation, interest, and regular study behaviors) have been associated to better performance and learning outcomes (Budé et al., 2007; Lavidas et al., 2020).

Math knowledge and psychological factors were assessed using validated psychometric scales. Specifically, basic mathematical skills relevant to an introductory Statistics course were assessed using the Mathematical Prerequisites for Psychometrics scale (Galli et al., 2008), with total scores obtained by summing the 30 dichotomously scored items. Academic motivation was assessed using the Relative Autonomy Index, proposed as an overall indicator of students’ motivational orientation in the context of The Academic Motivation Scale (Alivernini & Lucidi, 2008). Self-efficacy was assessed using the self-efficacy subscale of the Motivated Strategies for Learning Questionnaire (Bonanomi et al., 2018), which consists of nine items; the scale score was computed as the mean of the item responses. Finally, student engagement in statistics was assessed using the Student Engagement in Statistics scale (Whitney et al., 2019), which consists of 24 items; the scale score was computed as the mean of the item responses.

We fitted a multidimensional LC-IRT model on the dataset with the 30 ordinal indicators with three categories (fully correct, partially correct, and incorrect), where the latent dimensions were Knowledge, Application, and Judgment. The aim was to detect homogeneous subgroups of students in terms of latent ability according to the Dublin descriptors. Moreover, we investigated the influence of sex, math knowledge, academic motivation, self-efficacy, and engagement in statistics on students’ classification.

### Results

We selected the number of latent classes by jointly considering statistical fit, classification quality, class proportions, and substantive interpretability. We focused on non-trivial mixture solutions and estimated a sequence of multidimensional LC-IRT measurement models with C=2,…,4 classes. In all models, the latent factors were allowed to have class-specific variances and were specified as conditionally uncorrelated given latent class membership. Model fit indices and class proportions are reported in Table 8. The information criteria did not point to a single unequivocal solution. The Bayesian Information Criterion (BIC) favored the two-class solution, whereas the Akaike Information Criterion (AIC) reached its minimum for the three-class model. The four-class solution showed worse values on both AIC and BIC and was therefore not retained. Thus, the most relevant comparison was between the two- and three-class solutions. The three-class solution showed a higher entropy-based $R^{2}$ than the two-class solution, indicating improved classification quality. Substantively, the two-class model mainly distinguished students with higher versus lower statistical competence, whereas the three-class solution provided a more fine-grained and informative classification by distinguishing a high-performing profile, a mixed/intermediate profile, and a low-performing profile. Accordingly, although the three-class model was less parsimonious than the two-class model according to the BIC, it was retained because it provided the best balance between statistical fit, classification quality, class size, and substantive interpretability. This solution is also consistent with previous work on similar datasets (Fabbricatore et al., 2022, 2025).

**Table 8.** Fit Statistics and Class Proportions for the Multidimensional LC-IRT Measurement Models.

No. LC	Log-likelihood	BIC	AIC	No. par.	Entropy $R^{2}$	$\pi_{1}$	$\pi_{2}$	$\pi_{3}$	$\pi_{4}$
2	−7,485.41	15,695.20	15,224.82	127	.75	.50	.50	—	—
3	−7,412.36	15,914.14	15,206.72	191	.80	.29	.25	.46	—
4	−7,352.60	16,159.66	15,215.20	255	.83	.22	.14	.33	.31

*Note*. LC = latent class.

**Figure 10.**
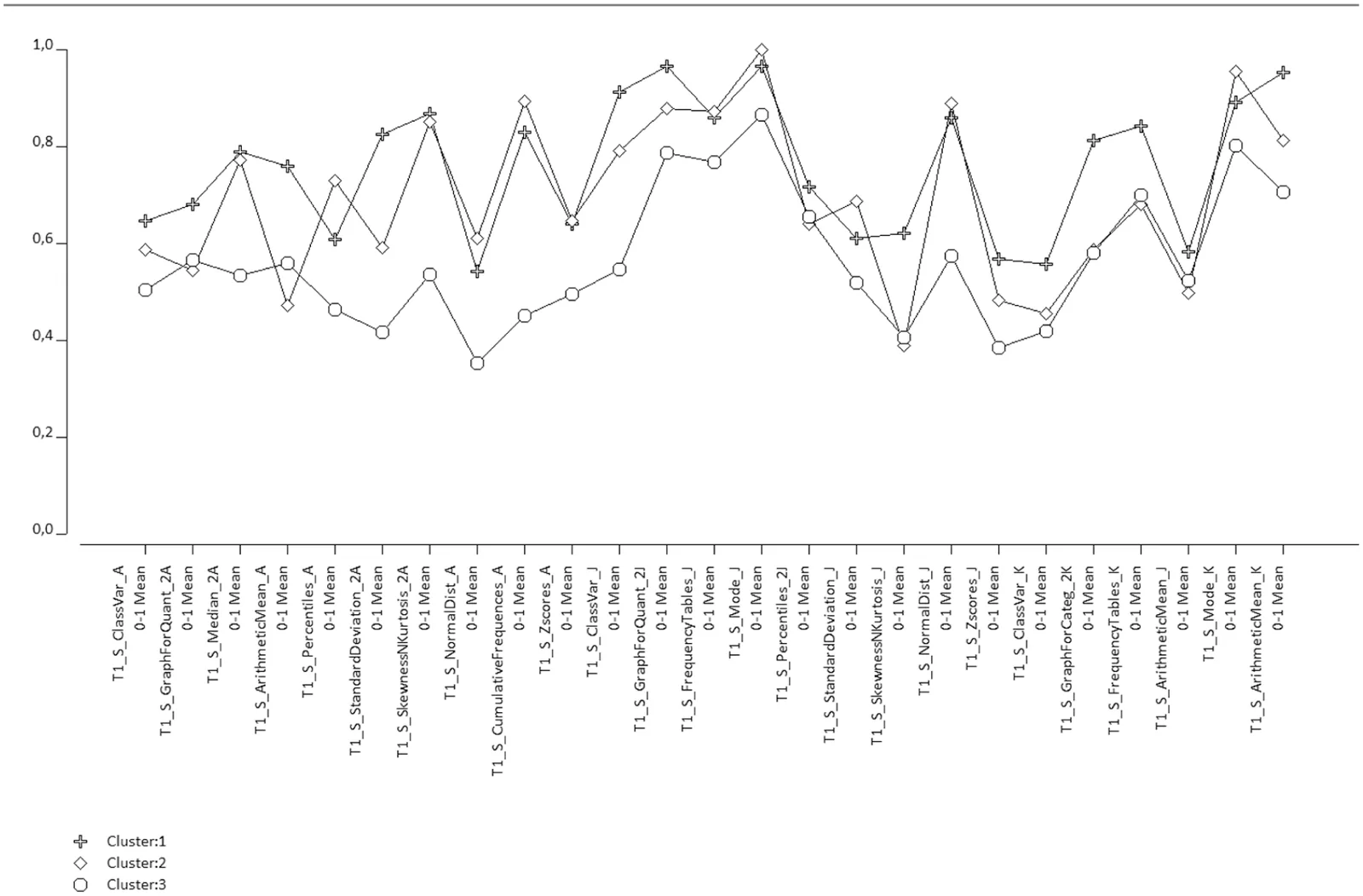
The class-specific response probabilities on the LC-IRT measurement model with 30 indicators on the real data example. *Note*. LC-IRT = latent class item response theory.

As seen in Figure 10, class 1 (29% of the respondents) comprises students who perform well on average on most items. Class 3, comprising 46% of the respondents, represents the lowest-performing group, and class 2 (25%) can be seen as a mixed performance group, excelling in some items and performing similarly to class 3 in some other items. Overall, students demonstrated a higher ability level in the Judgment dimension compared to both the Knowledge and Application dimensions. The measurement model parameters and all item loadings are reported in the Supplemental Material.

We fitted a structural model using the FIML and the two-step approach with five predictors, namely sex, math knowledge, academic motivation, self-efficacy, and engagement in statistics. Table 9 presents the parameter estimates of the structural model using dummy first coding, with class 1 as the reference category for the model estimated using the two-step and FIML approaches. The parameter estimates obtained with the two approaches lead to the same overall conclusions with regard to the direction of the effects, effect size, and significance. Sex emerged as a significant predictor, with females being more likely to belong to classes 2 and 3 (lower-performing classes). This finding is consistent with prior research showing gender differences in math-related subjects, where males tend to outperform females (Cipora et al., 2015; Else-Quest et al., 2010). Such differences are often discussed in terms of gender stereotypes, which frame math-related topics as predominantly male academic pursuits (Keller & Dauenheimer, 2003; Quinn & Spencer, 2001; Rodarte-Luna & Sherry, 2008). Math knowledge and self-efficacy were lower in class 3 compared to class 1, highlighting a significant difference between low-performers and top-performers. This aligns with previous studies emphasizing the foundational role of prior math knowledge in developing further STEM-related skills (Chiesi & Primi, 2010; Lavidas et al., 2020). Moreover, according to social cognitive theory and prior literature, belief in one’s capabilities (self-efficacy) seems to encourage students to engage with challenging subjects, leading to better performance (Bandura, 1977; Honicke & Broadbent, 2016; Judi et al., 2011; Rosli & Maat, 2017). Similarly, engagement in statistics is significantly associated with higher performance, as students in Class 1 exhibit greater engagement than those in Classes 2 and 3. This finding confirms that active participation, consistent study habits, and interest in the subject enhance learning outcomes—particularly in courses that non-STEM students often perceive as challenging (Budé et al., 2007; Fabbricatore et al., 2022; Lavidas et al., 2020). In contrast, academic motivation did not significantly predict performance, possibly because the measure captured general academic motivation rather than domain-specific motivation, resulting in a weaker effect on statistics performance compared to topic-specific psychological factors.

**Table 9.** Structural Model Parameters Using the Two-Step and FIML Estimation Approaches With Class 1 as Reference Class for the Real Data Example.

Term per cluster	Coef	*z*-Value	*p*-Value	Wald(0)	df	*p*-Value
Two-step						
Cl(2) 1	0.89	2.24	.02	28.51	2.00	< .001
Cl(3) 1	6.99	4.65	<.001			
Cl(2) Sex	0.99	2.24	.02	6.82	2.00	.03
Cl(3) Sex	0.90	2.15	.03			
Cl(2) Math knowledge	0.06	1.07	.29	11.71	2.00	<.001
Cl(3) Math knowledge	−0.09	−2.17	.03			
Cl(2) Academic motivation (RAI)	0.01	0.39	.69	2.16	2.00	.34
Cl(3) Academic motivation (RAI)	−0.01	−1.09	.27			
Cl(2) Self-efficacy	0.02	0.73	.46	9.07	2.00	.01
Cl(3) Self-efficacy	−0.06	−2.09	.04			
Cl(2) Engagement in statistics	−1.23	−2.87	<.001	8.81	2.00	.01
Cl(3) Engagement in statistics	−0.88	−2.25	.02			
FIML						
Cl(2) 1	1.19	0.54	.59	32.78	2.00	<.001
Cl(3) 1	9.71	4.83	<.001			
Cl(2) Sex	1.64	3.14	<.001	11.07	2.00	<.001
Cl(3) Sex	1.03	2.27	.02			
Cl(2) Math knowledge	0.05	0.74	.46	14.42	2.00	<.001
Cl(3) Math knowledge	−0.14	−2.56	.01			
Cl(2) Academic motivation (RAI)	0.017	0.92	.36	4.60	2.00	.10
Cl(3) Academic motivation (RAI)	−0.02	−1.16	.25			
Cl(2) Self-efficacy	0.01	0.34	.74	12.68	2.00	<.001
Cl(3) Self-efficacy	−0.09	−2.57	.01			
Cl(2) Engagement in statistics	−1.42	−2.95	<.001	9.48	2.00	.01
Cl(3) Engagement in statistics	−1.29	−2.54	.01			

*Note*. FIML = full information maximum likelihood; RAI = Relative Autonomy Index.

## Discussion

This study proposed and evaluated a two-step estimation approach for LC-IRT models, including both unidimensional and multidimensional specifications. Motivated by well-documented limitations of FIML estimation in complex mixture and IRT models, the proposed approach explicitly separates the estimation of the measurement model from the estimation of structural relations with covariates. This separation aligns with core principles in the SEM literature that emphasize the substantive interpretation of latent variables and the importance of guarding against interpretational confounding. A key strength of the proposed approach is its ability to preserve the meaning of the latent variables. By estimating the measurement model separately from the structural covariate model, and by incorporating DIF effects into the final measurement specification when needed, the two-step procedure helps preserve the substantive interpretation of the latent classes while avoiding confounding with structural covariate effects. This feature directly addresses concerns about interpretational confounding that arise in joint estimation frameworks and supports clearer substantive interpretation of both measurement and structural components.

The modular nature of the two-step approach also facilitates flexible modeling strategies. In particular, DIF effects can be introduced in a targeted manner after the latent structure has been established, avoiding the rapid proliferation of parameters that often accompanies fully specified FIML models. This flexibility is especially relevant in applied research settings, where sample sizes may be limited and model parsimony is essential. Moreover, the approach naturally extends to multidimensional LC-IRT models, where separating the recovery of latent profiles from their associations with covariates improves interpretability.

Across simulation conditions, the proposed two-step estimator demonstrated satisfactory parameter recovery for both measurement and structural parameters, particularly when classification uncertainty was adequately accounted for. In study 1 we had enough data to perform regression analysis predicting parameter bias, that showed that classification quality as measured by entropy, and estimation approach, and interaction of estimation approach and model misspecification are strong predictors of bias, namely FIML is more sensitive to misspecifications. In conditions with low class separation (low entropy), the two-step approach shows higher bias than the FIML approach.

With regard to coverage, it was generally adequate in conditions with good class separation, particularly when entropy is high. However, in low-entropy conditions, coverage deteriorated for the two-step estimator. This suggests that the standard errors from the second-step model are downward-biased, consistent with previous literature on stepwise estimation (Bakk & Kuha, 2018).

With regard to the real data application, we can conclude that the results are consistent with the two estimators, with the two-step approach having the advantage of easily modeling different types of step two modules. The three LCs show very different student competence profiles, and gender, math knowledge, self-efficacy and engagement with statistics emerge as significant predictors of belonging to the higher competence classes.

While the proposed two-step estimator demonstrated high efficiency and low bias across the majority of our simulated conditions, its performance remains strictly tied to the quality of the measurement model. Our results indicate that under low-entropy conditions—where class separation is weak or measurement precision is limited—the two-step approach is susceptible to substantial parameter bias. Furthermore, we observed a notable downward bias in standard errors in these suboptimal settings. These findings suggest that the two-step estimator is not a universal replacement for full-information approaches; rather, its utility is conditioned on achieving a well-defined measurement model. Consequently, when applying this stepwise framework to empirical data, we strongly recommend that researchers verify measurement quality through entropy and fit indices. Furthermore, to account for the observed downward bias in standard errors, it is essential that future implementations incorporate appropriate correction procedures, such as bootstrapping or robust variance estimators, to ensure valid statistical inference. As such, despite the advantages, several limitations should be acknowledged. First, as with all stepwise estimators, high levels of classification uncertainty may lead to efficiency losses or bias even after model correction—as such in situations with low entropy values, the FIML should be preferred. Second, while the present study focused on covariates and DIF effects, future work could explore extensions to more complex structural relations, such as latent interactions or nonlinear effects. Furthermore, we used the standard Hessian-based SE estimator that is default in software, so would be used most often in applied settings, while the SE estimator of the step-two model should be corrected for the amount of uncertainty in the step one model (Bakk & Kuha, 2018). While in models where the entropy is high the correction might not be necessary based on our results, in situations with low entropy it can be investigated if it improves model inference, even though the large parameter bias warrants against the use of stepwise methods in the situation of low entropy. Third, in the simulation studies, the number of latent classes was treated as known, and the systematic evaluation of class-enumeration performance was therefore left beyond the scope of the present work. Class enumeration is nevertheless a crucial issue in applied mixture modeling, and existing evidence provides partially different recommendations depending on the model and on the role of covariates. In factor mixture modeling, Wang et al. (2023) showed that including substantively meaningful covariates may improve class enumeration by increasing class separation. They also reported that the one-step estimator can yield better class enumeration than the three-step approach when class separation is poor, and can be more robust to misspecification or overfitting of direct covariate effects on factors. However, their study focused on direct covariate effects on factors and did not address direct covariate effects on observed indicators. In contrast, work on latent class models with covariates has shown that misspecified covariate effects during class enumeration may distort the selected number of classes, often leading to overextraction, and has recommended determining the number of latent classes before including covariates (Nylund-Gibson & Masyn, 2016; Vermunt, 2010). This recommendation is particularly relevant in the presence of DIF. Accordingly, in the context of stepwise latent class analysis in the presence of DIF, Vermunt and Magidson (2021) proposed a model-building strategy in which the number of classes is first determined using the class indicators only, after which potential DIF-inducing covariates are examined and the relevant direct effects are incorporated into the final step-one measurement model. Although this strategy was developed for three-step latent class analysis, it can also be adapted to two-step estimation settings. How this strategy performs for LC-IRT models under the proposed two-step estimation approach requires further investigation and represents an important direction for future research. Finally, although the simulations covered a range of realistic conditions, additional research is needed to assess performance under severe model misspecification or extreme class imbalance.

## Supplemental Material

sj-pdf-1-epm-10.1177_00131644261467757 – Supplemental material for A Two-Step Estimation Approach of Latent Class-Item Response Theory Models in the Presence of DIF and Multidimensionality Supplemental material, sj-pdf-1-epm-10.1177_00131644261467757 for A Two-Step Estimation Approach of Latent Class-Item Response Theory Models in the Presence of DIF and Multidimensionality by Zsuzsa Bakk and Rosa Fabbricatore in Educational and Psychological Measurement

## Notes

1. The simulation study is available on OSF: https://osf.io/he37c

2. It should be mentioned that the estimation time on average for a FIML and step one model was about an hour in Study 3, while for the step two model about 15 min. The time-efficiency of the stepwise estimation becomes relevant when multiple step two models are run for this complex multidimensional models.

3. Examples of the administered questions for each Dublin descriptor can be found in Fabbricatore et al. (2023, 2025).
