# Supplementary material for: A Two-Step Estimation Approach of Latent Class-Item Response Theory Models in the Presence of DIF and Multidimensionality
Source: Educ Psychol Meas. 2026 Aug 21:00131644261467757. Online ahead of print. doi: 10.1177/00131644261467757 (PMC13498696; doi:10.1177/00131644261467757)
Supplement: Supplementary file 1 [file sj-pdf-1-epm-10.1177_00131644261467757.pdf]

Supplementary material

Journal Title
XX(X):1–8
©The Author(s) 0000
Reprints and permission:
sagepub.co.uk/journalsPermissions.nav
DOI: 10.1177/ToBeAssigned
www.sagepub.com/
SAGE

Latent Gold syntax for Simulation 1

Below, the relevant parts of the latent GOLD syntax for Models A, B, C and D are presented. The relevant syntax of the step one model of the two-step approach is presented.

Model A

```
cluster <-1;
F1|cluster;
Y1 <-  (a1)1 | Cluster+ (1) F1;
y2 <-  (a2)1 | Cluster+ (1) F1;
y3 <-  (a3)1 | Cluster+ (1) F1;
y4 <-  (a4)1 | Cluster+ (1) F1;
y5 <-  (a5)1 | Cluster+ (1) F1;
```

Model B

```
cluster <-1+Z1;
F1|cluster;
Y1 <-  (a1)1 | Cluster+ (1) F1;
y2 <-  (a2)1 | Cluster+ (1) F1+Z1;
y3 <-  (a3)1 | Cluster+ (1) F1;
y4 <-  (a4)1 | Cluster+ (1) F1;
y5 <-  (a5)1 | Cluster+ (1) F1;
```

Model C

```
cluster <-1+Z1;
F1|cluster;
Y1 <-  (a1)1 | Cluster+ (1) F1;
y2 <-  (a2)1 | Cluster+ (1) F1+Z1|Cluster;
y3 <-  (a3)1 | Cluster+ (1) F1;
y4 <-  (a4)1 | Cluster+ (1) F1;
y5 <-  (a5)1 | Cluster+ (1) F1;
```

Model D

```
cluster <-1+Z2;
F1|cluster;
Y1 <-  (a1)1 | Cluster+ (1) F1;
y2 <-  (a2)1 | Cluster+ (1) F1+Z2;
```

```
y3 <-  (a3)1 | Cluster+ (1) F1;
y4 <-  (a4)1 | Cluster+ (1) F1;
y5 <-  (a5)1 | Cluster+ (1) F1;
```

Measurement model parameters of the real data example

Table 1. Class sizes of the multidimensional three class measurement model

Class	Coef	SE	Wald(0)	df
Cluster(2)	-0.17	0.23	10.25	2
Cluster(3)	0.45	0.18	–	–

Table 2. Continuous latent variables class specific variances by latent classes of the multidimensional LC-IRT measurement model for the real data example

Factor	Cluster	Coef	SE	Wald(0)	df
F1	Cluster(1)	0.28	0.10	7.86	3
F1	Cluster(2)	0.12	0.19	–	–
F1	Cluster(3)	0.09	0.11	–	–
F2	Cluster(1)	0.00	0.20	23.50	3
F2	Cluster(2)	0.00	0.32	–	–
F2	Cluster(3)	0.29	0.06	–	–
F3	Cluster(1)	0.22	0.17	3.49	3
F3	Cluster(2)	0.00	0.14	–	–
F3	Cluster(3)	0.13	0.09	–	–

Table 3. Application domain class specific item parameters for the real data example

Item	Cluster	Coef	SE	Wald(0)	df
T1_S_ClassVar_A(1)	Cluster(1)	1.71	0.50	35.25	6
T1_S_ClassVar_A(2)	Cluster(1)	1.42	0.52	–	–
T1_S_ClassVar_A(1)	Cluster(2)	0.25	0.40	–	–
T1_S_ClassVar_A(2)	Cluster(2)	0.76	0.42	–	–
T1_S_ClassVar_A(1)	Cluster(3)	0.47	0.23	–	–
T1_S_ClassVar_A(2)	Cluster(3)	0.02	0.25	–	–
T1_S_GraphForQuant_2A(1)	Cluster(1)	1.40	0.40	47.39	6
T1_S_GraphForQuant_2A(2)	Cluster(1)	1.50	0.41	–	–
T1_S_GraphForQuant_2A(1)	Cluster(2)	0.92	0.39	–	–
T1_S_GraphForQuant_2A(2)	Cluster(2)	0.35	0.44	–	–
T1_S_GraphForQuant_2A(1)	Cluster(3)	1.07	0.29	–	–
T1_S_GraphForQuant_2A(2)	Cluster(3)	0.55	0.30	–	–
T1_S_Median_2A(1)	Cluster(1)	-0.54	0.53	73.26	6
T1_S_Median_2A(2)	Cluster(1)	1.57	0.36	–	–
T1_S_Median_2A(1)	Cluster(2)	-5.63	5.68	–	–
T1_S_Median_2A(2)	Cluster(2)	1.24	0.37	–	–
T1_S_Median_2A(1)	Cluster(3)	-0.96	0.28	–	–
T1_S_Median_2A(2)	Cluster(3)	0.15	0.21	–	–
T1_S_ArithmeticMean_A(1)	Cluster(1)	-6.95	8.70	62.62	6
T1_S_ArithmeticMean_A(2)	Cluster(1)	1.22	0.32	–	–

Table 3. Application domain class specific item parameters for the real data example (continued)

Item	Cluster	Coef	SE	Wald(0)	df
T1_S_ArithmeticMean_A(1)	Cluster(2)	-3.13	0.93	–	–
T1_S_ArithmeticMean_A(2)	Cluster(2)	-0.12	0.29	–	–
T1_S_ArithmeticMean_A(1)	Cluster(3)	-1.66	0.36	–	–
T1_S_ArithmeticMean_A(2)	Cluster(3)	0.26	0.21	–	–
T1_S_Percentiles_A(1)	Cluster(1)	-2.59	0.85	49.96	6
T1_S_Percentiles_A(2)	Cluster(1)	0.48	0.27	–	–
T1_S_Percentiles_A(1)	Cluster(2)	0.16	0.53	–	–
T1_S_Percentiles_A(2)	Cluster(2)	1.31	0.46	–	–
T1_S_Percentiles_A(1)	Cluster(3)	-0.99	0.28	–	–
T1_S_Percentiles_A(2)	Cluster(3)	-0.17	0.21	–	–
T1_S_StandardDeviation_2A(1)	Cluster(1)	-1.72	0.93	82.49	6
T1_S_StandardDeviation_2A(2)	Cluster(1)	1.71	0.37	–	–
T1_S_StandardDeviation_2A(1)	Cluster(2)	-1.25	0.56	–	–
T1_S_StandardDeviation_2A(2)	Cluster(2)	0.42	0.30	–	–
T1_S_StandardDeviation_2A(1)	Cluster(3)	-1.50	0.31	–	–
T1_S_StandardDeviation_2A(2)	Cluster(3)	-0.40	0.21	–	–
T1_S_SkewnessNKurtosis_2A(1)	Cluster(1)	5.74	4.77	47.43	6
T1_S_SkewnessNKurtosis_2A(2)	Cluster(1)	6.78	4.77	–	–
T1_S_SkewnessNKurtosis_2A(1)	Cluster(2)	1.34	0.77	–	–
T1_S_SkewnessNKurtosis_2A(2)	Cluster(2)	2.68	0.71	–	–
T1_S_SkewnessNKurtosis_2A(1)	Cluster(3)	0.43	0.23	–	–
T1_S_SkewnessNKurtosis_2A(2)	Cluster(3)	0.24	0.26	–	–
T1_S_NormalDist_A(1)	Cluster(1)	-0.59	0.33	35.31	6
T1_S_NormalDist_A(2)	Cluster(1)	0.22	0.28	–	–
T1_S_NormalDist_A(1)	Cluster(2)	-0.48	0.45	–	–
T1_S_NormalDist_A(2)	Cluster(2)	0.55	0.35	–	–
T1_S_NormalDist_A(1)	Cluster(3)	-0.85	0.24	–	–
T1_S_NormalDist_A(2)	Cluster(3)	-0.82	0.24	–	–
T1_S_CumulativeFrequences_A(1)	Cluster(1)	0.35	0.50	74.75	6
T1_S_CumulativeFrequences_A(2)	Cluster(1)	2.09	0.43	–	–
T1_S_CumulativeFrequences_A(1)	Cluster(2)	-1.10	1.57	–	–
T1_S_CumulativeFrequences_A(2)	Cluster(2)	2.28	0.50	–	–
T1_S_CumulativeFrequences_A(1)	Cluster(3)	-0.51	0.23	–	–
T1_S_CumulativeFrequences_A(2)	Cluster(3)	-0.27	0.23	–	–
T1_S_Zscores_A(1)	Cluster(1)	0.56	0.34	17.80	6
T1_S_Zscores_A(2)	Cluster(1)	0.92	0.32	–	–
T1_S_Zscores_A(1)	Cluster(2)	1.33	0.48	–	–
T1_S_Zscores_A(2)	Cluster(2)	1.21	0.49	–	–
T1_S_Zscores_A(1)	Cluster(3)	0.05	0.23	–	–
T1_S_Zscores_A(2)	Cluster(3)	-0.04	0.24	–	–

Table 4. Judgement domain class specific item parameters for the real data example

Item	Cluster	Coef	SE	Wald(0)	df
T1_S_ClassVar_J(1)	Cluster(1)	-1.34	0.95	116.20	6
T1_S_ClassVar_J(2)	Cluster(1)	2.44	0.46	–	–
T1_S_ClassVar_J(1)	Cluster(2)	-1.71	0.81	–	–
T1_S_ClassVar_J(2)	Cluster(2)	1.39	0.39	–	–
T1_S_ClassVar_J(1)	Cluster(3)	-1.35	0.32	–	–
T1_S_ClassVar_J(2)	Cluster(3)	0.21	0.22	–	–
T1_S_GraphForQuant_2J(1)	Cluster(1)	-4.18	5.19	116.20	6
T1_S_GraphForQuant_2J(2)	Cluster(1)	3.31	0.70	–	–
T1_S_GraphForQuant_2J(1)	Cluster(2)	6.08	6.14	–	–
T1_S_GraphForQuant_2J(2)	Cluster(2)	7.21	6.14	–	–
T1_S_GraphForQuant_2J(1)	Cluster(3)	-0.16	0.35	–	–
T1_S_GraphForQuant_2J(2)	Cluster(3)	1.64	0.27	–	–
T1_S_FrequencyTables_J(1)	Cluster(1)	9.30	21.50	54.80	6
T1_S_FrequencyTables_J(2)	Cluster(1)	10.24	21.49	–	–
T1_S_FrequencyTables_J(1)	Cluster(2)	9.08	21.50	–	–
T1_S_FrequencyTables_J(2)	Cluster(2)	10.13	21.49	–	–
T1_S_FrequencyTables_J(1)	Cluster(3)	3.46	0.72	–	–
T1_S_FrequencyTables_J(2)	Cluster(3)	3.71	0.72	–	–
T1_S_Mode_J(1)	Cluster(1)	-5.47	13.66	123.65	6
T1_S_Mode_J(2)	Cluster(1)	3.33	0.59	–	–
T1_S_Mode_J(1)	Cluster(2)	-1.14	15.26	–	–
T1_S_Mode_J(2)	Cluster(2)	7.90	6.92	–	–
T1_S_Mode_J(1)	Cluster(3)	-1.06	0.52	–	–
T1_S_Mode_J(2)	Cluster(3)	2.09	0.29	–	–
T1_S_Percentiles_2J(1)	Cluster(1)	0.19	0.39	60.80	6
T1_S_Percentiles_2J(2)	Cluster(1)	1.23	0.33	–	–
T1_S_Percentiles_2J(1)	Cluster(2)	-0.72	0.48	–	–
T1_S_Percentiles_2J(2)	Cluster(2)	0.67	0.32	–	–
T1_S_Percentiles_2J(1)	Cluster(3)	0.43	0.28	–	–
T1_S_Percentiles_2J(2)	Cluster(3)	0.98	0.27	–	–
T1_S_StandardDeviation_J(1)	Cluster(1)	-2.01	0.60	63.29	6
T1_S_StandardDeviation_J(2)	Cluster(1)	0.47	0.26	–	–
T1_S_StandardDeviation_J(1)	Cluster(2)	-0.56	0.50	–	–
T1_S_StandardDeviation_J(2)	Cluster(2)	0.93	0.37	–	–
T1_S_StandardDeviation_J(1)	Cluster(3)	-1.00	0.29	–	–
T1_S_StandardDeviation_J(2)	Cluster(3)	0.09	0.22	–	–
T1_S_SkewnessNKurtosis_J(1)	Cluster(1)	1.14	0.39	21.03	6
T1_S_SkewnessNKurtosis_J(2)	Cluster(1)	0.96	0.39	–	–
T1_S_SkewnessNKurtosis_J(1)	Cluster(2)	1.19	0.32	–	–
T1_S_SkewnessNKurtosis_J(2)	Cluster(2)	-0.88	0.47	–	–
T1_S_SkewnessNKurtosis_J(1)	Cluster(3)	-0.43	0.23	–	–
T1_S_SkewnessNKurtosis_J(2)	Cluster(3)	-0.57	0.25	–	–
T1_S_NormalDist_J(1)	Cluster(1)	-1.02	0.79	90.17	6

Table 4. Judgement domain class specific item parameters for the real data example (continued)

Item	Cluster	Coef	SE	Wald(0)	df
T1_S_NormalDist_J(2)	Cluster(1)	1.94	0.48	–	–
T1_S_NormalDist_J(1)	Cluster(2)	1.78	1.16	–	–
T1_S_NormalDist_J(2)	Cluster(2)	3.36	1.13	–	–
T1_S_NormalDist_J(1)	Cluster(3)	-0.48	0.28	–	–
T1_S_NormalDist_J(2)	Cluster(3)	0.39	0.24	–	–
T1_S_Zscores_J(1)	Cluster(1)	-2.71	0.96	27.41	6
T1_S_Zscores_J(2)	Cluster(1)	0.28	0.25	–	–
T1_S_Zscores_J(1)	Cluster(2)	0.69	0.35	–	–
T1_S_Zscores_J(2)	Cluster(2)	-0.14	0.42	–	–
T1_S_Zscores_J(1)	Cluster(3)	-0.61	0.23	–	–
T1_S_Zscores_J(2)	Cluster(3)	-0.68	0.26	–	–
T1_S_ArithmeticMean_K(1)	Cluster(1)	-4.79	5.82	54.44	6
T1_S_ArithmeticMean_K(2)	Cluster(1)	0.33	0.25	–	–
T1_S_ArithmeticMean_K(1)	Cluster(2)	-1.89	0.60	–	–
T1_S_ArithmeticMean_K(2)	Cluster(2)	-0.01	0.30	–	–
T1_S_ArithmeticMean_K(1)	Cluster(3)	-2.05	0.41	–	–
T1_S_ArithmeticMean_K(2)	Cluster(3)	0.10	0.21	–	–

Table 5. Knowledge domain class specific item parameters

Item	Cluster	Coef	SE	Wald(0)	df
T1_S_ClassVar_K(1)	Cluster(1)	1.81	0.48	45.07	6
T1_S_ClassVar_K(2)	Cluster(1)	0.72	0.51	–	–
T1_S_ClassVar_K(1)	Cluster(2)	-0.10	0.34	–	–
T1_S_ClassVar_K(2)	Cluster(2)	-0.28	0.32	–	–
T1_S_ClassVar_K(1)	Cluster(3)	0.38	0.22	–	–
T1_S_ClassVar_K(2)	Cluster(3)	-0.68	0.27	–	–
T1_S_GraphForCateg_2K(1)	Cluster(1)	0.78	0.53	74.72	6
T1_S_GraphForCateg_2K(2)	Cluster(1)	2.10	0.48	–	–
T1_S_GraphForCateg_2K(1)	Cluster(2)	-0.38	0.38	–	–
T1_S_GraphForCateg_2K(2)	Cluster(2)	0.44	0.31	–	–
T1_S_GraphForCateg_2K(1)	Cluster(3)	-1.39	0.37	–	–
T1_S_GraphForCateg_2K(2)	Cluster(3)	0.36	0.20	–	–
T1_S_FrequencyTables_K(1)	Cluster(1)	1.16	0.60	94.37	6
T1_S_FrequencyTables_K(2)	Cluster(1)	2.53	0.55	–	–
T1_S_FrequencyTables_K(1)	Cluster(2)	-5.92	4.67	–	–
T1_S_FrequencyTables_K(2)	Cluster(2)	0.75	0.29	–	–
T1_S_FrequencyTables_K(1)	Cluster(3)	-0.15	0.30	–	–
T1_S_FrequencyTables_K(2)	Cluster(3)	1.07	0.24	–	–
T1_S_Mode_K(1)	Cluster(1)	1.64	1.00	149.80	6
T1_S_Mode_K(2)	Cluster(1)	3.31	1.00	–	–
T1_S_Mode_K(1)	Cluster(2)	-4.55	6.00	–	–

Table 5. Knowledge domain class specific item parameters (continued)

Item	Cluster	Coef	SE	Wald(0)	df
T1_S_Mode_K(2)	Cluster(2)	3.02	1.11	–	–
T1_S_Mode_K(1)	Cluster(3)	-0.50	0.40	–	–
T1_S_Mode_K(2)	Cluster(3)	1.62	0.29	–	–
T1_S_Percentiles_K(1)	Cluster(1)	-1.10	0.38	20.15	6
T1_S_Percentiles_K(2)	Cluster(1)	-0.53	0.29	–	–
T1_S_Percentiles_K(1)	Cluster(2)	-0.57	0.39	–	–
T1_S_Percentiles_K(2)	Cluster(2)	0.07	0.31	–	–
T1_S_Percentiles_K(1)	Cluster(3)	-0.38	0.24	–	–
T1_S_Percentiles_K(2)	Cluster(3)	-0.35	0.23	–	–
T1_S_StandardDeviation_K(1)	Cluster(1)	5.77	5.44	63.51	6
T1_S_StandardDeviation_K(2)	Cluster(1)	7.16	5.43	–	–
T1_S_StandardDeviation_K(1)	Cluster(2)	0.83	0.46	–	–
T1_S_StandardDeviation_K(2)	Cluster(2)	1.61	0.41	–	–
T1_S_StandardDeviation_K(1)	Cluster(3)	0.84	0.27	–	–
T1_S_StandardDeviation_K(2)	Cluster(3)	1.08	0.27	–	–
T1_S_SkewnessNKurtosis_K(1)	Cluster(1)	2.63	0.76	59.58	6
T1_S_SkewnessNKurtosis_K(2)	Cluster(1)	3.44	0.74	–	–
T1_S_SkewnessNKurtosis_K(1)	Cluster(2)	7.36	8.07	–	–
T1_S_SkewnessNKurtosis_K(2)	Cluster(2)	7.84	8.07	–	–
T1_S_SkewnessNKurtosis_K(1)	Cluster(3)	1.66	0.33	–	–
T1_S_SkewnessNKurtosis_K(2)	Cluster(3)	1.67	0.33	–	–
T1_S_NormalDist_K(1)	Cluster(1)	0.92	0.64	123.07	6
T1_S_NormalDist_K(2)	Cluster(1)	2.86	0.55	–	–
T1_S_NormalDist_K(1)	Cluster(2)	5.51	5.15	–	–
T1_S_NormalDist_K(2)	Cluster(2)	7.32	5.14	–	–
T1_S_NormalDist_K(1)	Cluster(3)	-0.22	0.29	–	–
T1_S_NormalDist_K(2)	Cluster(3)	1.04	0.23	–	–
T1_S_Zscores_K(1)	Cluster(1)	-0.72	11.03	99.52	6
T1_S_Zscores_K(2)	Cluster(1)	8.05	6.26	–	–
T1_S_Zscores_K(1)	Cluster(2)	-4.68	9.13	–	–
T1_S_Zscores_K(2)	Cluster(2)	3.43	0.77	–	–
T1_S_Zscores_K(1)	Cluster(3)	-0.62	0.38	–	–
T1_S_Zscores_K(2)	Cluster(3)	1.63	0.26	–	–

Real data example measurement model. Loadings and R^2

Table 6. Loadings and R^2

Loadings	F1	F2	F3	Cluster	R^2
T1_S_ClassVar_A	0.10	-0.00	-0.00	0.17	0.04
T1_S_GraphForQuant_2A	0.10	-0.00	-0.00	0.16	0.04
T1_S_Median_2A	0.11	-0.00	-0.00	0.28	0.09
T1_S_ArithmeticMean_A	0.13	-0.00	-0.00	0.23	0.07
T1_S_Percentiles_A	0.13	-0.00	-0.00	0.24	0.08
T1_S_StandardDeviation_2A	0.11	-0.00	-0.00	0.37	0.15
T1_S_SkewnessNKurtosis_2A	0.07	-0.00	-0.00	0.45	0.21
T1_S_NormalDist_A	0.13	-0.00	-0.00	0.25	0.09
T1_S_CumulativeFrequences_A	0.09	-0.00	-0.00	0.48	0.24
T1_S_Zscores_A	0.11	-0.00	-0.00	0.19	0.05
T1_S_ClassVar_J	-0.00	0.13	-0.00	0.37	0.17
T1_S_GraphForQuant_2J	-0.00	0.12	-0.00	0.25	0.09
T1_S_FrequencyTables_J	-0.00	0.07	-0.00	0.19	0.05
T1_S_Mode_J	-0.00	0.11	-0.00	0.24	0.08
T1_S_Percentiles_2J	-0.00	0.09	-0.00	0.08	0.03
T1_S_StandardDeviation_J	-0.00	0.11	-0.00	0.15	0.05
T1_S_SkewnessNKurtosis_J	-0.00	0.11	-0.00	0.26	0.09
T1_S_NormalDist_J	-0.00	0.12	-0.00	0.37	0.17
T1_S_Zscores_J	-0.00	0.10	-0.00	0.18	0.05
T1_S_ClassVar_K	-0.00	-0.00	0.07	0.17	0.03
T1_S_GraphForCateg_2K	-0.00	-0.00	0.09	0.24	0.07
T1_S_FrequencyTables_K	-0.00	-0.00	0.08	0.17	0.04
T1_S_ArithmeticMean_J	-0.00	0.12	0.00	0.07	0.04
T1_S_Mode_K	-0.00	-0.00	0.08	0.20	0.05
T1_S_ArithmeticMean_K	-0.00	-0.00	0.07	0.31	0.10
T1_S_Percentiles_K	-0.00	-0.00	0.10	0.10	0.03
T1_S_StandardDeviation_K	-0.00	-0.00	0.06	0.30	0.10
T1_S_SkewnessNKurtosis_K	-0.00	-0.00	0.07	0.22	0.06
T1_S_NormalDist_K	-0.00	-0.00	0.08	0.31	0.11
T1_S_Zscores_K	-0.00	-0.00	0.06	0.32	0.11
